# Status of primary and secondary mental healthcare of people with severe mental illness: an epidemiological study from the UK PARTNERS2 programme

**DOI:** 10.1192/bjo.2021.10

**Published:** 2021-02-15

**Authors:** Siobhan Reilly, Catherine McCabe, Natalie Marchevsky, Maria Green, Linda Davies, Natalie Ives, Humera Plappert, Jon Allard, Tim Rawcliffe, John Gibson, Michael Clark, Vanessa Pinfold, Linda Gask, Peter Huxley, Richard Byng, Max Birchwood

**Affiliations:** Division of Health Research, Lancaster University, UK; and Centre for Applied Dementia Studies, Faculty of Health Studies, University of Bradford, UK; McPin Foundation, UK; Birmingham Clinical Trials Unit, Birmingham University, UK; Division of Health Research, Lancaster University, UK; Division of Population Health, Health Services Research and Primary Care, University of Manchester, UK; Birmingham Clinical Trials Unit, Birmingham University, UK; Institute for Mental Health, School of Psychology, University of Birmingham, UK; Cornwall Partnership NHS Foundation Trust, UK; and Community and Primary Care Research Group, Faculty of Medicine, University of Plymouth, UK; Division of Health Research, Lancaster University, UK; Institute for Mental Health, School of Psychology, University of Birmingham, UK; London School of Economics and Political Science, UK; McPin Foundation, UK; Division of Population Health, Health Services Research and Primary Care, University of Manchester, UK; Centre for Mental Health and Society, School of Health Sciences, Bangor University, UK; Community and Primary Care Research Group, Faculty of Medicine, University of Plymouth, UK; Warwick Medical School, University of Warwick, UK

**Keywords:** Primary healthcare, community mental healthcare, severe mental illness, service utilisation, continuity of care

## Abstract

**Background:**

There is global interest in the reconfiguration of community mental health services, including primary care, to improve clinical and cost effectiveness.

**Aims:**

This study seeks to describe patterns of service use, continuity of care, health risks, physical healthcare monitoring and the balance between primary and secondary mental healthcare for people with severe mental illness in receipt of secondary mental healthcare in the UK.

**Method:**

We conducted an epidemiological medical records review in three UK sites. We identified 297 cases randomly selected from the three participating mental health services. Data were manually extracted from electronic patient medical records from both secondary and primary care, for a 2-year period (2012–2014). Continuous data were summarised by mean and s.d. or median and interquartile range (IQR). Categorical data were summarised as percentages.

**Results:**

The majority of care was from secondary care practitioners: of the 18 210 direct contacts recorded, 76% were from secondary care (median, 36.5; IQR, 14–68) and 24% were from primary care (median, 10; IQR, 5–20). There was evidence of poor longitudinal continuity: in primary care, 31% of people had poor longitudinal continuity (Modified Modified Continuity Index ≤0.5), and 43% had a single named care coordinator in secondary care services over the 2 years.

**Conclusions:**

The study indicates scope for improvement in supporting mental health service delivery in primary care. Greater knowledge of how care is organised presents an opportunity to ensure some rebalancing of the care that all people with severe mental illness receive, when they need it. A future publication will examine differences between the three sites that participated in this study.

The health of people with severe mental illness (SMI) is a global problem, with physical health disparities resulting in high personal, social and economic burden across the lifespan.^1^ People with schizophrenia or bipolar disorder have poorer physical health,^2^ with multiple physical comorbidities and healthcare risks,^3^ including greater risk of cardiovascular disease^4^ and a significantly lower life expectancy that the general population.^5,6^ There is a widespread view that mental health problems should be tackled at the primary care level in high- and low-income countries.^7^ In England, the Quality and Outcomes Framework (QOF) is a financial incentive scheme that aims to reward general practices for delivering good-quality care.^8^ However, this financial incentive has ended for people with SMI, despite evidence of an increase in the frequency of monitoring in primary care and increase in the identification of physical comorbidities.^9,10^

For many people with SMI, their primary and secondary mental healthcare is being delivered by separate and largely unconnected teams. This has a negative impact on longitudinal continuity of care.^11^ This is an ongoing pattern of healthcare interaction that occurs in the same place, with the same medical record and professionals, so that there is a growing knowledge of the patient by those providing the care.^12^ In recent years, there have been financial and policy drivers in England to encourage more people previously supported by community mental health teams (CMHTs) to be discharged to primary care; for example, a financial imperative driven by austerity cuts to funding and moves to implement a payment-by-results model (based on allocating patients to specific care clusters based on their needs, and then intended to relate to care packages). This policy was later renamed as the National Tariff Payment System,^13^ and is a policy driver that sets out the aspiration to deliver what is thought to be a better approach to care. A decade ago, in the PARTNERS1 study, we found that approximately 31% of people with SMI in the UK were seen only in the primary care setting.^14^ The study reported in this paper was conducted as part of the first phase of the National Institute for Health Research (NIHR)-funded PARTNERS2 research programme (International Standard Randomised Controlled Trials Number: ISRCTN95702682).^15^ We aimed to define the current status of integration and collaboration post-QOF, as well as identifying where the strengths and weaknesses lie, to inform better long-term solutions. Therefore, in this study, we address three key questions. First, what is the current level of primary care and secondary mental healthcare contact for those individuals with SMI maintained in secondary care? Second, what is the level of longitudinal continuity of care within primary and secondary care? And finally, what health risks were recorded and what physical healthcare monitoring was undertaken?

## Method

### Design, sites and sampling frame

This multi-site, cross-sectional epidemiological review of primary and secondary care contacts was conducted in three locations across England. Three host National Health Service (NHS) Trust sites, reflecting a geographical spread across England, were invited to participate in this programme of research. The research team (which includes service user researchers, two of whom are co-authors) worked with these Trusts to select secondary care mental health teams to reflect urban/rural and deprivation-level diversity. We approached the Clinical Commissioning Groups responsible for locally commissioning healthcare services, and invited them and relevant practices to participate. Ethical approval for the study was obtained from the National Research Ethics Service Committee – West Midlands (approval number 14/WM/0052); data collection was deemed to be service development, therefore patient consent was not required. The study adheres to the international reporting standards for observational studies,^16^ and has public and patient involvement embedded throughout the whole programme.^17^

#### Inclusion and exclusion criteria

Those eligible for the study were on the CMHT case-load, registered at a participating general practitioner (GP) practice, had a clinical diagnosis of bipolar disorder or schizophrenia and were aged ≥18 years. Those within care clusters 11–17 who did not have evidence of recent psychosis (within the past 24 months) and who had a confirmed diagnosis outside of our eligibility criteria were excluded. Care clusters provide a framework for planning and organising mental health services, care and support that can be provided for individuals linked to the payment-by-results model.

#### Sampling

**Fig. 1:**
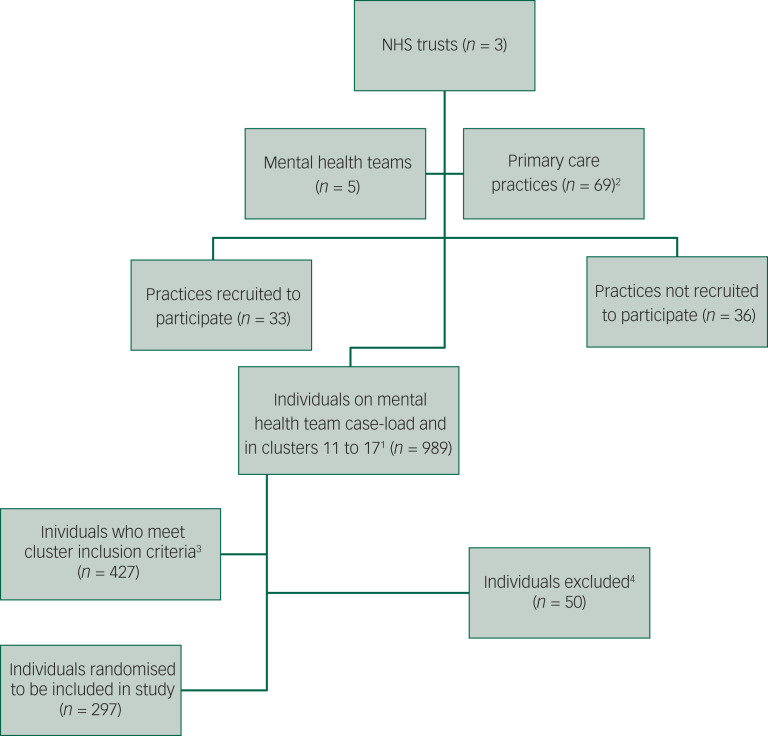
Flow chart of steps for identifying the sample for this study. ^1^Cases were included in the study if patients had been clustered within care clusters 11–17 at any point during the 2-year data extraction period, therefore, it is possible that the most recent cluster may not have been a psychosis cluster (https://improvement.nhs.uk/documents/485/Annex_DtE_Mental_health_clustering_tool.pdf). In mental health there are 21 clusters that cover a range of diagnosis and needs. Cluster 11 represents those with ongoing/recurrent psychosis (low symptoms) and cluster 12 is for those with ongoing/recurrent psychosis (high disability). To overcome the possibility of some misclassifications in the clusters, the clinical members of the research team (R.B. and L.G.) reviewed any individual cases where there was confusion about confirmed or appropriateness of diagnosis/misclassification or borderline cases. ^2^ See Supplementary Table 1, which compares participating practices with practices not included by practice list size, number of general practitioners and index of multiple deprivation. Participating practices tended to have a larger number of general practitioners and were located within less deprived areas compared with the national average. ^3^At any point during data extraction period 1 September 2012 to 31 August 2014. ^4^Exclusions were for not having a confirmed diagnosis of schizophrenia, psychosis, bipolar disorder or associated spectrum diagnoses. NHS, National Health Service.

We worked with five CMHT, 33 participating GP practices, and team administrators and performance management teams in each NHS Trust to identify individuals who met the inclusion criteria on 1 September 2014 (Fig. 1). We stratified practices in each of the sites, according to the number of eligible individuals registered with the practice that were on the mental health team case list, i.e. small (0–4), medium (5–19) and large (≥20) practices. Cases eligible for inclusion were selected by proportional stratified random sampling (based on the proportion of all eligible individuals in each stratum). We aimed to identify 100 randomly selected eligible cases from the three participating mental health services.

### Data collection and data entry

We developed structured survey tools to collect data from both secondary care teams and GP practices. We manually extracted data from electronic patient secondary mental healthcare and primary care medical records, using specially developed data extraction tools developed from the tools used in our previous study, PARTNERS1.^14^ We sought public and patient involvement in the development of these tools through our Lived Experience Advisory Panels. The data extraction tools were piloted on four sets of records in each site in both primary and secondary care, for feasibility. The data reported in this paper are summarised in Supplementary Figure 1 available at https://doi.org/10.1192/bjo.2021.7. The data extraction tools and detailed manual are available in Supplementary File 1.

Data were collected for each set of records included in the analysis (*n* = 297) from both secondary and primary healthcare teams’ electronic records by research staff (between October 2014 and June 2016). The data collected related to a 2-year period (2012–2014). Patient identifiable data were not collected and care records were given unique identifiable numbers. Non-identifiable completed data extraction tools were transferred securely, in accordance with local data protection policies, to the research team for data entry. Data were checked randomly for concordance and fidelity; data collectors had weekly discussions to standardise data collection between sites. Data were entered by PARTNERS2 research staff onto a database designed by the Primary Care Clinical Research and Trials Unit at the University of Birmingham.

### Measures

Longitudinal continuity for GP contact within primary care was measured with the Modified Modified Continuity Index (MMCI). This measures the number of GPs seen; a higher continuity score occurs when there are larger numbers of visits with a smaller number of GPs^18^ (see also Supplementary Fig. 1). Calculation of longitudinal continuity of primary care was restricted to individuals with a minimum of three contacts. Poor GP continuity was defined as an MMCI of ≤0.5. Longitudinal continuity for secondary mental healthcare was measured as the proportion of individuals with a one named care coordinator continuing over the 2 years, and the number of different psychiatrists seen per person over the 2 years.

### Analysis

Analyses were conducted with Stata software, version 13 (Stats-Corp) for Windows. Participating practices were compared with those not participating and all practices in England on list size, number of GPs and Index of Multiple Deprivation.^19^ Descriptive statistics and measures of variance were derived relating to individual demographics; number and type of medications; number of comorbidities; direct service contacts (defined as face-to-face or telephone contacts between an individual and a health or social care professional); reasons for contacts; and continuity of care, including type and frequency of contacts with primary and secondary care, proportions of individuals that have no contact with primary care and time between contacts in primary and secondary care. We also present descriptive health outcome data for individuals overall. Continuous data were summarised by mean and s.d., or median and interquartile range if data were skewed. Categorical data were summarised as percentages.

## Results

After describing the setting and the sample characteristics, we have organised the results according to our three key questions.

### Setting: practice and teams

Thirty-three of the sixty-nine practices (48%) approached consented to participate, and data were extracted from the case records of 297 individuals from these practices (Fig. 1). Participating practices tended to have a larger number of GPs and were located within less deprived areas, compared with the national average (Supplementary Table 1).

### Sample characteristics

Of the 297 individuals included in the study, the average age was 47 years and 56% were male (Table 1). A total of 33% were from Black and minority ethnic groups (15% Asian, 12% Black and 6% mixed ethnicity), but almost a quarter of ethnicity data were missing. Over a third lived alone (36%). Just 10% were recorded as in employment. Around half (53%) were smokers and 16% were ex-smokers; smoking cessation advice was reported to have been given to 66% of the smokers. The most recent diagnosis of the majority of the sample was schizophrenia (57%) or bipolar disorder (21%), and a quarter of individuals had evidence of a dual diagnosis (substance or alcohol misuse). Approximately two-thirds (66%) had been most recently allocated to either cluster 11 or 12 (referring to, respectively, people with ‘ongoing recurrent psychosis (low symptoms)’ and ‘ongoing or recurrent psychosis (high disability)’.

**Table 1:** Characteristics of total patient cohort: sociodemographics, most recent SMI diagnosis, cluster, number and type of medications taken, and physical conditions

Characteristic^a^	*n* (%), *n* = 2971
Age	Mean [s.d.]	47.4 [12.0]
Gender	Male	167 (56)
	Missing	1 (<1%)
Ethnicity^b^	White	128 (43)
	Black and minority ethnic	97 (33)
	Missing	72 (24)
Receiving benefits	Yes	223 (75)
	Missing	30 (10)
Living situation	Alone	106 (36)
	Lives with spouse/family	133 (45)
	Non-family, group home or other	55 (19)
	Missing	3 (1)
Employment	Paid employment	29 (9)
	Not working/other	261 (88)
	Missing	7 (2)
Most recent SMI diagnosis	Schizophrenia	170 (57)
	Bipolar disorder	61 (21)
	Other^c^	65 (22)
	Missing	1 (<1%)
Smoking status	Smoker	157 (53)
	Missing	9 (3)
Smokers given cessation advice	Yes	104 (66)
	Missing	19 (12)
Alcohol drinkers	Yes	156 (53)
	Missing	12 (4)
Recreational drug use	Yes	48 (16)
	Missing	15 (5)
Evidence of a dual diagnosis	Yes	75 (25)
	Missing	21 (7)
Entitled to Section 117 (Mental Health Act 1983) aftercare	Yes	49 (16)
	Missing	177 (60)
Subject to a community treatment order	Yes	21 (7)
	Missing	187 (63)
Most recent cluster	Care cluster 11: ongoing/recurrent psychosis (low symptom)	122 (41)
	Care cluster 12: ongoing/recurrent psychosis (high disability)	73 (25)
	Care cluster 13: ongoing/recurrent psychosis (high symptom and disability)	40 (13)
	Care clusters 14–17^d^	28 (9)
Number of types of medications taken^e^		*n =* 295
	Median {IQR}	2 {1–3}
Types of medication^f^	Atypical antipsychotic	237 (80)
	Antidepressant medication	107 (36)
	Conventional antipsychotic	74 (25)
	Bipolar disorder medication	72 (24)
	Anti-anxiety medication	80 (27)
	Other medication	64 (22)
Number of other physical health conditions^g^		*n =* 295
	Median {IQR}	1 {0–2}
	0	103 (35)
	1	111 (37)
	≥2	81 (27)

SMI, severe mental illness; IQR, interquartile range.

a. The majority of these data were taken from the primary care *pro forma*. However, where there was no data for a particular variable on the primary care form and there was data in the secondary care form, the secondary care data was used, to minimise the amount of missing data.

b. Ethnicity: there was a high level of missing data as this was only collected adequately in one site (Birmingham); however, the missing data is likely to represent a high proportion of White individuals.

c. Other diagnoses included schizotypal personality disorder, persistent delusional disorder, acute/transient psychotic disorder, induced delusional disorder, schizoaffective disorder, severe depression with psychosis and other.

d. Care cluster 14–17: 14 (psychotic crisis), 15 (severe psychotic depression), 16 (dual diagnosis – substance misuse and mental illness) and 17 (psychosis and affective disorder – difficult to engage). Care clusters provide a framework for planning and organising mental health services, care and support that can be provided for individuals linked to the payment-by-results model.

e. Types of medication include conventional antipsychotics, atypical antipsychotics, bipolar disorder medications, antidepressant medications, antianxiety medications, other mental health medications and any other medication.

f. People can receive more than one type of medication, therefore percentages can add up to more than 100%. Bipolar disorder medications included (see Supplementary File 1) carbamazepine, gabapentin, lamotrigine, lithium carbonate, lithium citrate, valproic acid and topimarate.

g. Physical conditions include diabetes, asthma, chronic obstructive pulmonary disorder, epilepsy, hypertension, stroke, thyroid disorder, ischaemic heart disease, heart failure, chronic kidney disease, learning disability, hearing problems, rheumatoid arthritis, cancer, osteoarthritis, obesity, visual problems and other.

### What is the current level of primary care and secondary mental health care contact for those people with SMI maintained in secondary care?

#### Patterns of secondary mental healthcare use, hospital admissions and discharging back to primary care

**Fig. 2:**
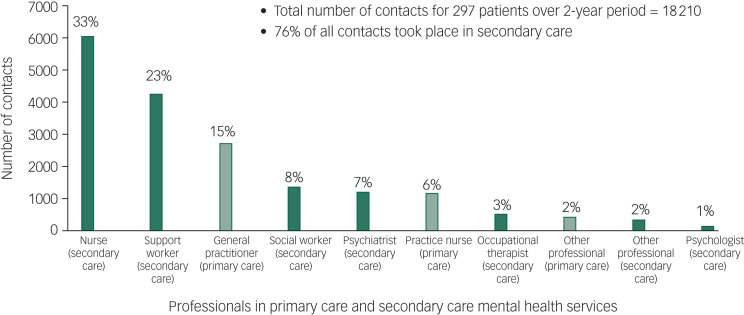
Number (and percentage) of direct patient contacts with professionals in primary care and secondary mental healthcare during the 2-year period.

Around a fifth of individuals did not have any contact with a psychiatrist (22%). Similarly, 25% did not have any contact with a community psychiatric nurse over the 2-year period. The majority (88%) of individuals had one or more contacts with a secondary mental healthcare professional. The median contacts with a psychiatrist was 3 (interquartile range (IQR), 1–6) and median contacts with a nurse was 12 (IQR, 0–35). The total number of direct patient-related contacts with secondary mental healthcare were 13 910 (median, 36.5; IQR, 14–68). This represented 76% of the total 18 210 direct contacts recorded across primary and secondary care (Fig. 2). A further 1369 (8.9%) contacts were recorded as ‘no access visit, did not attend, failed contact’. The median number of days between secondary care contacts was 13 days (IQR, 7–22) (Table 2).

**Table 2:** Direct contacts with professionals providing care in primary and secondary care mental health services, different professionals seen and longitudinal continuity of care

	*n* (%), *N* = 297	Median {IQR}	Range
Secondary mental healthcare and different professionals seen			
Number of no access visits/did not attend/failed contacts	1369		
Total direct patient contacts^a^	13 910		
Number of patients with direct patient contacts	292 (98%)	36.5 {14–68}	0–208
Number of patients with one or more direct contact	260 (88%)		
Psychiatrist contacts^b^	233 (78%)	3 {1–6}	0–27
Nurse contacts	217 (73%)	12 {0–35}	0–148
Social worker contacts	110 (37%)	0 {0–4}	0–95
Occupational therapist contacts	61 (21%)	0 {0–0}	0–55
Psychologist contacts^c^	24 (8%)	0 {0–0}	0–20
Support worker contacts	155 (52%)	1 {0–18}	0–146
Other professional contacts^d^	61 (21%)	0 {0–0}	0–70
Primary care and different professionals seen			
Total direct patient contacts^a^	4300		
Directs contacts (all patients with non-missing data)	279	10 {5–20}	0–109
Number of patients with one or more direct contact	273 (92%)		5–499
Total number of direct contacts per patient			
GP contacts	256 (86%)	6 {2–13}	0–88
GPs seen per patient		3 {1–5}	0–25
Longitudinal continuity of care for GP contacts (MMCI^e^ *N* = 205), mean (s.d.)	0.58 (0.27)		
% Poor longitudinal continuity of care (MMCI < 0.5)	31		
Nurse contacts	198 (67%)	2 {0–5}	0–49
Hospital admissions			
Total number of admissions^f^	297		
Mental health admissions^g^^,^^h^	185	0 {0–1}	0–8
Patients with one or more mental health admissions	79		
Non-mental health admissions	88	0 {0–0}	0–8
Patients with one or more non-mental health admissions	45		
Length of hospital stay per patient (in patients with one or more admission)	107		
Median of each patient's mean length of stay for mental health admissions^i^		23 {6–49}	

IQR, interquartile range; GP, general practitioner; MMCI, Modified Modified Continuity Index.

a. Contacts where the type of professional seen is missing (*n* = 261 secondary care; *n* = 13 primary care) have not been included.

b. Psychiatrist includes consultant psychiatrist and trust/staff psychiatrist (including junior psychiatrist).

c. Psychologist includes clinical psychologist and assistant psychologist.

d. Other secondary care professional includes social worker assistant, occupational therapist assistant, healthcare assistant, peer worker, voluntary sector worker, student, administrator, police doctor and other mental health worker in secondary care.

e. Longitudinal continuity of care was measured with the MMCI, calculated as follows: MMCI = (1 – number of different GPs seen/number of contacts with a GP)/(1 – 1/number of contacts with a GP). This measure relates to a patient's number of contacts with a health provider (e.g. GP practice) to the number of different professionals seen across those contacts (e.g. different GPs). In primary care, if all of a patient's contacts were with the same GP, then MMCI = 1; if they were all with different GPs, then MMCI = 0. Calculation of longitudinal continuity of primary care was restricted to individuals with a minimum of three GP contacts (*n* = 205).

f. Patients with no hospital admissions data have been assumed to have had no hospital admissions during the data extraction period.

g. In-patient records were accessed for data regarding mental health admissions (in-patient contacts were not included in the contact count). The research team did not have access to in-patient records from general hospitals.

h. Reasons for admissions: request for psychiatric help (154 contacts, 76 patients), physical health problem (59 contacts, 32 patients), suicide attempt/overdose (30 contacts, 15 patients), specialist investigation (11 contacts, 7 patients), self-harm (6 contacts, 5 patients), alcohol/substance misuse (3 contacts, 3 patients), accidental injury (7 contacts, 5 patients), diagnosis (1 contact, 1 patient) and other (13 contacts, 10 patients).

i. Calculated using only patients with at least one hospital admission.

Over a quarter of individuals (79; 27%) had a mental health admission over the 2 years; 45 (15%) had a non-mental health admission. The median length of (any) hospital stay per person was 23 days (IQR, 6–49).

Thirty-seven (12%) individuals had been discharged to primary care within the 2-year period, but were included on the secondary care case-load when the sample was taken. Of these, 22 (59%) were discharged back to primary care more than once: nine were discharged twice , seven were discharged three times, four were discharged four times, one was discharged five times and one was discharged seven times.

#### Patterns of primary healthcare use

A high proportion of individuals (44%) had seven or more GP contacts, 25% had three to six GP contacts and 17% had one or two GP contacts. There were 7% of cases that did not have any contact with a GP, and 6% were missing data. The median number of contacts with GPs was 6 (IQR, 2–13; range, 0–88). Over a quarter (28%) had one or two contacts with a nurse. Around a fifth (21%) had three to six contacts and 18% had seven or more. There were 27% that did not have any contact with a nurse, and 6% were missing data. The median number of contacts with nurses was 2 (IQR, 0–5). The median number of direct primary care contacts was 10 (IQR, 5–20), with a total of 4300 across the 2 years (Table 2).

The majority of GP contacts (72%) were in practice contacts, 27% were telephone contacts; 91% of contacts with nurses were in practice contacts whereas 8% were telephone contacts. The majority of primary care contacts were at the practice (72%) or by telephone (27%). The majority of direct contacts were with a GP (63%), 27% were with a nurse and 10% were with another health professional. Around a third (34%) of contacts were for mental health/ongoing mental health conditions including physical health effects as a result of mental health, and a third (33%) were for physical health/ongoing physical health conditions (see Supplementary Table 2). The median number of days between primary care contacts was 45 (IQR, 27–82).

### What is the level of longitudinal continuity of care within primary and secondary care?

#### Longitudinal continuity of care

Longitudinal continuity of secondary care: less than a half of individuals had a one named care coordinator continuing over the 2 years (43%). The median number of different psychiatrists seen per individual over the 2-year period was 2 (IQR, 1–3), and ranged from 0 to 14. Almost a third (31%) had poor longitudinal continuity of primary care (MMCI *<* 0.5) (mean, 0.58; s.d. 0.27).

### What health risks were recorded and what physical healthcare monitoring was undertaken?

#### Health conditions and monitoring

Around two-thirds of the sample (65%) had another major health morbidity recorded; over a quarter (27%) had another two or more major health morbidities recorded. The median number of medications currently prescribed was 2 (IQR, 1–3); 80% were prescribed atypical antipsychotic medications. During the 2-year period, there was evidence of the following: one or more physical health checks for blood pressure in 87% (*n* = 257; median, 2; IQR, 1–4) of individuals, body mass index in 79% (*n* = 236; median, 1; IQR, 1–2) of individuals, cholesterol in 65% (*n* = 193; median, 1; IQR, 0–2) of individuals and haemoglobin A1c in 39% (*n* = 115; median, 0; IQR, 0–1) of individuals. There were 45 (15%) individuals who had one or more electrocardiograms. Just under a quarter of women had one or more cervical smear tests (31/129; 24%), and less than a third of women who were aged ≥50 years at the start of the data extraction period had one or more mammograms (18/61; 30%).

## Discussion

### Summary of findings

This study suggests that for individuals with SMI who are in contact with secondary mental health services, these services are centrally involved in their care. Three-quarters of all direct contacts recorded across primary and secondary care were from secondary care. Individuals were seen on average every 2 weeks by specialist care practitioners, although with much variability, and yet only around four in ten individuals had one named care coordinator continuing over the 2 years. Three-quarters of all contacts were with either a nurse or support worker. In contrast, these individuals were also seen on average every 6 weeks in primary care, and almost a third had poor continuity of care in relation to contacts with a GP. GPs accounted for 15% of all contacts, whereas psychiatrists accounted for 7% of all contacts. Less than a tenth (9%) of contacts were for health-promoting activities. Women's health checks were lower than the general population, and only 1% of primary care contacts were recorded as being for social, family, housing, employment or financial reasons. Three-quarters of individuals were in receipt of benefits, only a tenth were in employment, a quarter reported substance or alcohol misuse and around two-thirds (65%) had another major health morbidity. Individuals failed to attend for just under a tenth of secondary care contacts. Of those who were discharged to primary care within the 2-year period, a high proportion (59%) of these had one or more repeat discharges from secondary mental health care.

### Findings in the context of previous research

In our original cross-sectional epidemiological review of the primary care records of 1150 patients with bipolar disorder or schizophrenia,^9^ we found that only the details of psychiatrists’ contacts were recorded in primary care. Of those who were seen in secondary care (796/1150; 69%), the majority of these (61%) had no more than two secondary care contacts per year that were recorded in their primary care records. In our original study, only 64 of 796 (8%) of participants had evidence in their primary care records of a contact with a community psychiatric nurse, whereas in this study, 217 of 297 (73%) participants had evidence of at least one contact with a CMHT nurse. The original study reported only what was known in primary care, thus representing a potential 65% underestimate of the actual contacts with a CMHT nurse. Only the contacts with the psychiatrist tend to get reported to primary care; this is because out-patient doctors routinely write to GPs after each consultation, whereas community key workers, who see patients more frequently, do not.^20^ This gap in knowledge is likely to have negative implications for patient safety. Primary care is unlikely to be so much ‘in the dark’ for other patient groups in receipt of specialist care.

The annual primary care consultations rates for this group approximates to 7.7 for 1 year, if the rate of consultations is constant over the 2-year period. This is slightly higher than the annual rate for the general population for the same time period (5.16 per year).^21^ Primary care consultation rates appear to be lower in our study compared with previous studies of individuals with SMI. The number of direct primary care contacts (median of 10) over 24 months in the present study compared with: i) our earlier notes review (mean annual face-to-face consultation rate for individuals with SMI with GPs and nurses of 6.7, for 2008/2009^14^; ii) a longitudinal cohort study reported annual mean face-to-face primary care contacts as 10.9 (s.d. 12)^10^; and iii) another study that reported on cardiovascular disease treatment for SMI recorded a mean of 9.4 primary care consultations (s.d. 8) over 9 months.^20^ These studies are more inclusive, including individuals who were not seen in secondary care, so the lower rates of primary care contacts in our study may be explained by the fact that we have focused only on individuals who are seen in secondary care. The high secondary care contact rates may be explained through mechanisms for substitution. For example, where secondary care personnel prevent the need for primary care or where routine monitoring/testing, medication management, and patient education promotes self-management.

Longitudinal continuity in primary care was poor for 31% of patients, significantly higher than the 21% found in our earlier study^14^ conducted in 2010. The increasing rates of poor continuity of care may also be explained by the fact that we have focused only on individuals who are seen in secondary care. However, they have further negative implications for patient safety. A recent systematic review has shown that increased continuity of care by doctors is associated with lower mortality rates.^21^ Furthermore, frequent changes in staff providing care for people with psychosis are associated with poorer quality of care^22^ and worse clinical outcomes.^11^ Turnover of staff (particularly psychiatrists) may account for poorer continuity of secondary care. It is important to note that we have defined continuity of care by contacts with a care coordinator and having the same person allocated over the study period. It is possible that the lack of contact with a care coordinator reported for a substantial number of patients may be explained by differing service structures and configurations, as well as services using different case-loads and skill mixes to meet needs. For example, some CMHTs had access to non-clinically qualified support workers for this group, who had supervision from a qualified team member who was a care coordinator.

Over two-thirds of the sample (65%) had two or more conditions compared with 24% of people in England.^23^ There was evidence that health checks were being carried out, which may be because our data was collected during the period before the removal of cardiometabolic QOF indicators in 2014 (requiring annual recording of weight, blood cholesterol and glucose). Further research is required to assess the long-term impact of removing SMI indicators from the QOF.^10^ As half of individuals did not have any health promotion activity recorded as the reason for primary healthcare contacts, it is likely that health promotion is likely to be lower than needed for this group. Opportunities for opportunistic health promotion and addressing physical healthcare needs are being missed despite the high levels of need, and our data suggests that nurses in primary care are underused for this group. Furthermore, we found that only 24% of eligible female patients had had a smear test. This is 48% lower than the national rate of 72% of eligible women (aged 25–64 years) who were recorded as screened adequately.^24^ Furthermore, lower rates of mammograms are consistent with the wider literature, which indicates that women with schizophrenia and other psychosis are about half as likely as the general population to receive mammography screening.^25^, ^26^, ^27^ About two-thirds of smokers had been given smoking cessation advice. We did not specifically collect information on nutrition or exercise.

### Strengths and weaknesses

This study has several strengths. This study focuses upon individual level data from both primary and secondary healthcare records to observe service use across the primary–secondary care interface in multiple sites. Our study is both timely and, to our knowledge, is the first UK study to comprehensively explore how care is delivered for individuals with SMI. The nature of the data collection methods and the embedded study within a larger programme of research meant that the study was limited to three sites, however, this has allowed for a rich tapestry of data relating to frequency, health professionals seen, locus of care and continuity of care. Although time consuming, manual data extraction from patient healthcare records was the only logistical way we could obtain this information. Consistent data collection was made possible by having clearly specified collection procedures in a detailed study manual, weekly discussions to standardise data collection and checking data randomly for concordance and fidelity.

In terms of limitations, this study relies on routinely collected data from both primary and secondary care electronic record systems, which can, but does not always, result in problems of incompleteness, interpretation and imperfections. As with all studies that retrospectively extract data from clinical records, the quality of the data reported is dependent on what is available to be captured across different electronic or paper medical records. This may vary with different healthcare systems and cultures. Any information about third-sector involvement in individual's care or support is extremely limited, only being recorded when their involvement was coordinated with or observed by a secondary care worker. In addition, the information available to distinguish between different GPs was derived from the GP initials. These were variably reported, so the statistician had to make assumptions to estimate a count of number of different GPs seen, which was used to calculate the MMCI. We did not capture the time involved with each patient contact, nor were we able to capture when individuals failed to attend in primary care. We also did not obtain any data on the views of individuals on their healthcare needs or who should provide care to them. This study took place in three NHS Trusts, five mental health teams and thirty-three practice teams; the overall mean size of practices was similar to practices nationally. However, compared with the national average, the practices participating tended to have a larger number of GPs and were located in more affluent areas in two of the sites; thus, caution is needed when generalising the findings to other geographical areas. Furthermore, as involvement with secondary mental health teams was an inclusion criterion this study does not include a significant proportion of patients with SMI (33%) that are managed in primary care only.^14^ We plan a further publication where we will compare the level of primary care and secondary mental healthcare contact for individuals with SMI maintained in secondary care between the three sites. We will also compare the longitudinal continuity of care within primary and secondary care and the costs of primary care and secondary mental healthcare contact.

### Implications

There are three key implications. First, this study demonstrates that the biggest workload is borne by secondary care mental health services. Second, there were high variations in care received by those included in this study. We know from comparing these results to those in our previous study^14^ that an imbalance in care within this group is highlighted; those with SMI who are managed only in primary care receive far less intervention than most of those managed in secondary care. Policy makers, commissioners and clinicians should seek to redress the imbalance, ensuring that all those with SMI receive excellent quality care. Third, when the results of this study are compared with previous evidence where data has been collected in primary care,^14^ the information held in primary care hugely underestimates the amount of care received by most of this group. This has implications for continuity of care, collaborative working and integrated care. A barrier to good care is a lack of appropriate data-sharing, which would enable organisations to identify comorbidities, anticipate problems and plan care in a holistic fashion.^28^A lack of integrated information systems means that primary care is largely unaware of the extensive input from secondary mental healthcare.

The poor continuity of care observed in this study was consistent with declining continuity of care over a decade.^11^ The configuration of services and organisation of care is a huge determinant of continuity of care and health outcomes.^11,24^ Our study supports the current UK policy toward providing connected mental and physical healthcare. In these new models of care, the current levels of intensity of contacts are not likely to be sustainable for the majority of individuals with schizophrenia and bipolar disorder, although frequency could be sustained or enhanced for the those currently seeing secondary care practitioners infrequently, in a new integrated model. This coupled with the high levels of poor continuity of care with regards to their physical health provides a clear signal for more collaborative care models and shared data records. Discharge to primary care might also be more feasible and safer if patients are subsequently followed up and supported through a system of collaborative care.^29^ Indeed, this study is part of the PARTNERS2 study, where we are developing and testing a collaborative care model for people with SMI to examine whether a new service based in primary care is better than existing care for people with SMI, as assessed by changes in quality of life. We are also updating the corresponding Cochrane Collaboration systematic review.^29^

We need good-quality data for decision-making, particularly in this time of service pressures and restructuring. Having greater knowledge of how care is delivered presents an opportunity for commissioners and those responsible for delivering services, to ensure that all people with SMI receive the care they need when they need it. However, replicating this study would be difficult and costly; future patient record systems need to acknowledge the need to generate this type of data. Clinical Commissioning Groups are tasked with providing high quality specialised mental health services that are integrated with local health systems and are delivered as close to home as possible.^30^ Incentive schemes that are set at a local level have more ability to flex around the local population's needs and reduce health inequalities. The findings in this study pertain to England, but have relevance to other countries considering how best to configure care services for people with SMI.

Our study provides data to help national and local policy makers make decisions on how to structure and potentially rebalance primary and secondary mental healthcare services for people with SMI.^31^ We recommend that the healthcare system is changed to support improvement in health service delivery for people with SMI. More specifically, commissioners could establish regional quality improvement contracts that have targets for mental health; configure specialist mental health services so that they are integrated within primary healthcare, and can address issues of continuity of care by having a key worker who can help to organise the care of people with SMI; and ensure both specialist and primary healthcare information systems are integrated, to facilitate informational continuity. Further, clinicians and practitioners should systematically identify people with SMI and follow up within primary care to ensure that all can easily access effective specialist mental healthcare when they need it. Also, mental health services and primary care should make efforts to facilitate and maintain continuity of care whenever possible. NHS England and NHS Improvement need to ensure that staff are trained, supported and incentivised to provide opportunistic health promotion and address physical healthcare needs for this group; and ensure that the problem of high staff turnover rates and high rates of job vacancies is addressed, so that it does not affect the care of this group. Finally, researchers should systematically evaluate these new models of care, using rigorous research methods.

## Data Availability

Data is not available because of ethical restrictions. Because of the nature of this research, which was deemed to be service development, individuals whose records were analysed in this study were not approached to seek agreement for their data to be shared publicly, so supporting data is not available.

## Supplementary material

For supplementary material accompanying this paper visit http://dx.doi.org/10.1192/bjo.2021.10. click here to view supplementary material
