# Supplementary material for: Status of primary and secondary mental healthcare of people with severe mental illness: an epidemiological study from the UK PARTNERS2 programme
Source: BJPsych Open. 2021 Feb 15;7(2):e53. doi: 10.1192/bjo.2021.10 (PMC8058911; doi:10.1192/bjo.2021.10)
Supplement: Supplementary file 1 [file S2056472421000107sup001.zip › ONLINE_DET_Manualforsecondarycare.docx]

PARTNERS2: Assessment of local care pathways and current services for people with severe mental

illness

## OBSERVATIONAL RETROSPECTIVE COHORT STUDY AT 1^ST^ JULY 2014 _________________________________________________________________________________

SECONDARY CARE MANUAL

If you have any questions, please contact the Research Assistant in your area:

Contents

Introduction 3

Study background and aims 4

Study procedures 5

Guide to completing data extraction form 6

Appendices 20

**Introduction**

The aim of the study is to enable primary care and community based mental health services to work more closely together and to develop a model of care within which selected individuals with severe mental illness (SMI) can be managed in primary care with secondary care support.

The aim of this initial piece of research is to describe the process of current care for service users with serious mental illness, and to help us to better target those who could benefit from primary/secondary collaborative care and assess any potential risk and safety issues. We will do this by undertaking a review of 100 current service users and 30 discharged service users in three regions (Birmingham, Lancashire and Devon) and observe their care pathways of over the previous 24 months. In addition to this, we will also be gathering these data from secondary mental health care and primary care medical records for the same service users. The pathway data will include health service use (consultations with different health professionals), admissions (routine and emergency), discharge to and referral back from primary care.

This manual is intended as a guide and reminder of study procedures and requirements. Please take the time to have a look through this manual and familiarise yourself with the study details before starting work. It is also likely that you will need to refer back to the manual during the course of the study to ensure the data collected is accurate. This is important as the way in which data is collected needs to be consistent across all three sites.

| **Inclusion criteria are:**     - The service user has been clustered in clusters 11- 17 within the previous 24 months (as identified by the secondary care notes) - Is currently on the caseload of the CMHT or - Has been discharged from the CMHT during the previous 12 months - Is registered with a general practice that is participating in the study - Does not meet any of the exclusion criteria (Please see SOP on selecting eligible patients for data collection for further details) |
| --- |

You can contact Humera Khan, programme manager, on 0121 414 6947 or use the contact details of the research assistants on the front page if you want to talk through the study procedures and have any questions about starting work on the study.

Finally, thank you very much for undertaking this work. With your help ultimately the study will facilitate development of an integrated care model that works within UK primary care to improve the health outcomes of people with SMI.

**Study Background**

PARTNERS2 focuses on the estimated 70% of adults with schizophrenia or bipolar disorder who are currently seen and treated in primary care alone, or in a limited way by CMHTs. We propose to develop and test a new way of working with this group - collaborative care. Collaborative care is a novel system of delivering care, with a case manager working in and in collaboration with primary care under specialist supervision, which provides a platform for provision of evidence-based psychological, social and pharmacological intervention. The study involves a number of different methods including a notes review exercise, focus groups, structured survey, telephone interviews, systematic literature reviews and finally a randomized controlled trial.

We mainly want your help with the first phase of work – the notes review. We have selected a CMHT or equivalent in each locality and have obtained approvals for their linked GP practices. We would like you to select 100 individuals fulfilling the eligibility criteria (page 3) and extract data using the form providing.

You should have received the following documents in your data collection pack:

1. A data extraction form for 100 service users and some extra copies
2. A study manual (this document)
3. A patient identifier sheet (to be kept by you)

If you have not received any of these documents, please call Humera Khan (0121 414 6947) or the research assistant within your area.

| **Study Aims**  1. What is the current state of primary care and secondary mental health care for the individuals with serious mental illness in a rapidly evolving NHS?  2. What are the key components of primary care based collaborative care for people with serious mental illness?  3. What are the barriers and facilitators to implementing this model of care in an evolving commissioning environment?  4. How acceptable is collaborative care to patients, carers, practitioners, commissioners and managers?  5. What is the impact of primary care based collaborative care for people with serious mental illness on processes, social inclusion, health outcomes, and cost effectiveness of care within the constraints of a pilot trial? |
| --- |

**Study procedures**

1. **Selection of CMHTs and GP Practices**

A member of the study team will work with CSOs and CMHTs to identify eligible practices and gain consent from the GP practices to take part in the study. Letters will be sent from the clinical team to GPs saying that we are undertaking this research and CSOs from PCRN will collect data from primary care.

1. **Selection of eligible service users**

Eligible service users will be identified through their secondary care notes. Please see SOP on selecting eligible service users for data collection for more information. The PARTNERS 2 research assistant in your locality will liaise with you about which services users have been randomised for data collection for each GP practice and will send you a patient identifier sheet that will determine:

- The unique identifier number
- The corresponding NHS number for each patient
- The dates for which data should be collected

This patient identifier sheet should be kept safe for future reference in case there is a query in respect of the data. This should be retained by you in a secure place, such as locked filing cabinet or within a secure folder electronically. **It is essential that this document is not lost during the duration of the study,** as it will be the only way to identify individuals included in the study. The researchers will not have a copy of this list.

1. **Data collection**

Data for the notes review will be extracted by RSOs or by research assistants with the appropriate contracts with the trust. The next section will provide more detailed guidance on how to complete the data extraction form. Please familiarise yourself with both the questionnaire and this manual before undertaking any data extraction.

Data extraction forms are anonymous as patient identifiable information in not collected, however, please keep completed forms in a secure location within a locked cabinet and liaise with the PARTNERS 2 research assistant in your local area about returning these.

If it is not possible to store these in a secure location within a locked cabinet then please contact the local PARTNERS 2 research assistant.

**Thank you for your help and commitment to the PARTNERS2 study. If you would like any further information, please use the contact details on the front page.**

**Guide to completing data extraction form (Secondary care)**

**Introduction**

As this is an England-wide study and there is no standard computer system used by all teams across England, it is not possible in this manual to give precise instructions with regard to how the computer system that you will be using. If you do not feel confident in undertaking this step or any other described in this manual and there is no-one within the team who can assist you, please contact a member of the research team (contact details given at the front of manual) or your local research assistant before proceeding.

In the next section there is detailed guidance on how to complete each question. Please familiarise yourself with the data extraction tool and manual before undertaking any data extraction. It is possible that when you come to complete a data extraction form you find that the patient does not meet our inclusion criteria.

It needs to be emphasised that it is very unlikely that you’ll be able to complete the data extraction form in a linear fashion as the relevant information in the patients’ notes will be recorded differently for each patient and in each computer system. The more familiar you become with the data extraction form the easier it will be to complete so before you start please take the time to look at the types of information we require so you can skip to the relevant section when you find the information in the notes. For example, 1 attachment letter from a psychiatrist might contain information on medicines, diagnosis, CPA review and care coordinators and we ask about these in different sections of the data extraction form.

Sometimes the information we are requesting per patient are in many ways aspirational, and we recognise the very real problems in obtaining the information. If information cannot be located, this is an important finding in itself. But we do not wish to report that omissions have been made when in fact they haven’t, so accuracy with whether or not data is actually missing is vital too.

**If there are any sections of this form which do not work for your practice please contact us immediately before you undertake the extraction of data.**

Please complete all the boxes. In the event of you not being able to obtain certain information (we hope this won’t happen too often), please fill the boxes with 99 (or the boxes in the first line of a table), so that we know you have not merely forgotten or missed any items. The form requires you to fill in a number of dates. If however, any of these are not applicable, please fill the relevant boxes with ‘/’s. It is important for us to know the difference between missing data (‘99’s) and that which is not applicable (‘/’s) as this will have a significant impact on the findings.

As we are trying to collect data from within a 2 year period, it may be necessary for you to look at previous referrals on the system.

1. **Your details**

**Q.1 Name of Data Extractor**

Please complete the name of the person extracting the data

1. **Patient ID /Data extraction dates/Site details**

**Q2.** **Unique patient study number**.

Please record the unique patient study number for each patient as was provided to you on the patient identifier sheet.

The unique study numbers in Birmingham will start with 1001, 1002 etc.

The unique study numbers in Devon will start with 2001, 2002 etc.

The unique study numbers in Lancashire will start with 3001, 3002 etc.

The numbers refer to should be retained in case any follow up / data checking is required. This should be completed and retained by you in case there are any queries in respect of the data. It should be kept in a secure place (e.g. a locked filing cabinet if paper list, or within a secure folder on a PC if electronic list). It is essential that this document is not lost during the duration of the study, as it will be the only way to identify patients included in the study. The researchers will not have a copy of this list.

**Q3.** **Date of data extraction**

Please record the date that you collected the data.

**Q4. Site number**

Please record the site number using the codes

1 = Birmingham

2 = Devon

3 = Lancashire

**Q5. Data extraction period**

Please record the dates that the data extraction period commenced and ended (dd/mm/yyyy). This should be available on the patient identifier sheet provided to you by the PARTNERS 2 Research Assistant. If this is not provided, please contact the research assistant to find out which dates you should use.

This may vary between patients (based on discharge dates), however must be the same dates for both each patient in primary and secondary care data collection tools.

**Q6. GP practice Code**

Please see appendix 3 for the GP practice codes in your locality.

**Q7. GP practice postcode:**

Please record the postcode of the GP practice.

**C Patient demographic details**

Apart from DOB some of these questions may be difficult to complete unless your patients complete a new patient questionnaire which contains these details. In many cases you might come across the information in a discharge letter or in a social services assessment form. Please go back to complete the information if you do find it.

On eCR, some of this data (such as ethnicity, current living situation, employment, benefits and religion) may be found within the patient ‘details’ section.

**Q.8** **Patient age (in years)**

This is the age of the patient (in years) at 31^st^ August 2014 – which is the end of the data extraction period we are interested in.

**Q 9. Patient gender**

Please record whether the patient is male, female or transgender by ticking the appropriate box.

**Q10. Is this patient British?**

Please record whether the patient is British or not, or tick not recorded if this information is not available.

**Q11. Patient ethnicity**

This information may not be recorded routinely on the system you are using. If it is, you may come across the information contained within other documents such as discharge letters, assessments within the patient’s notes or within the physical health modules.

**Q 12. Current living situation**

This may be an important factor related to severity of illness. Again the patient’s living situation may not be routinely recorded but may be hidden within documents and attachments (e.g. letters) contained within the patient’s notes. Please come back to this question if you locate the information later on.

Notes: Please code the patient’s usual living situation here. If the patient is currently in respite care, please record their usual living situation here but record the respite care in the ‘admissions’ question - which is question 34.

Notes: If a patient is residing in supportive housing or a residential home please record this as ‘group home’

**Q13. Does the patient have any children?**

Please record whether or not the patient has any children, if this information is available.

If the patient does have children, please specify the number within each age range in the boxes provided.

**Q 14. Has this patient been homeless in the last 5 years?**

This information is unlikely to be recorded routinely but if you do come across this information please tick the appropriate box.

**Q 15. Current employment**

This information may be recorded within one of the assessment documents. Please tick the appropriate box if you find this information.

**Q16. Religion**

Please record the patient’s religion if this information is available.

**Q 17. Does this patient currently receive any benefits including permitted work, personal independence payments, employment support allowance, or disability allowance?**

This information may be recorded in one of the assessment documents, or may be within one of the letters. Please tick the appropriate box if you do come across this information.

**Q 18. Does this patient have refugee status?**

This information is unlikely to be recorded routinely but if you do come across this information please tick the appropriate box.

**Q 19. Does this patient have asylum seeker status?**

Again, this information is unlikely to be recorded routinely but if you do come across this information please tick the appropriate box.

**D Patient health and medication details**

**Q 20. What is this patient’s current (most recent) serious mental illness diagnosis?**

There may be a diagnosis section in which you might find this information. Please record the most recent diagnosis that is recorded, even if this is not prior to the data collection period.

**Q21. Further information on diagnosis:**

Please provide as much additional details on diagnoses as reasonably possible (e.g. exact diagnosis, previous diagnoses, year of first serious mental illness diagnosis).

For example, the most recent diagnosis for the service user may be schizoaffective disorder so you would record this for question 20, however they may have had a previous diagnosis of schizophrenia in a previous year so please record this here with the year of diagnosis if available.

Please use the codes provided to record the exact diagnosis as documented in the notes.

Any historical data (even if estimated) is important here.

**Q 22. What date was this diagnosis (Q 20) first made?**

A thorough assessment of the patient’s full history will be necessary for this question. This is with any psychiatric care agency in any area.

In some circumstances you may need to give your best guess from looking at hospital letters. You may need to go back through previous referrals if available to find out this information. On eCR you may find this information on some of the assessments such as health and social needs assessments.

**Q23. Please specify whether this date is an exact date or an estimate.**

Please indicate whether the date that this diagnosis was first made is an estimate or an exact date.

**Q 24. Is there evidence of a dual diagnosis? (i.e. substance misuse or alcohol problem)**

Please record whether or not the patient has a **current** diagnosis of dual diagnosis as well as serious mental illness.

**Q 25. Have any cluster category been recorded within the data extraction period?**

Please record whether or not any cluster categories have been recorded between the specified dates.

If there has been cluster categories recorded between the specified dates, please record all of these in the table provided, including the code of the category, the date it was recorded and whether this was in the specified timing for the re-assessment.

| 1 | Common mental health problems (Low severity and disability) | 12 weeks |
| --- | --- | --- |
| 2 | Common mental health problems | 15 weeks |
| 3 | Non psychotic (Moderate severity) | 6 months |
| 4 | Non psychotic (Severe) | 6 months |
| 5 | Non psychotic (Very severe) | 6 months |
| 6 | Non psychotic disorders of overvalued ideas | 6 months |
| 7 | Enduring non-psychotic disorders of overvalued ideas | Annual |
| 8 | Non psychotic chaotic and challenging disorders | Annual |
| 9 | Blank Cluster | N/A |
| 10 | First episode in psychosis | Annual |
| 11 | Ongoing recurrent psychosis (low symptoms) | Annual |
| 12 | Ongoing or recurrent psychosis (high disability) | Annual |
| 13 | Ongoing or recurrent psychosis (high symptom and disability) | Annual |
| 14 | Psychotic Crisis | 4 weeks |
| 15 | Severe psychotic depression | 4 weeks |
| 16 | Dual diagnosis – substance misuse and mental illness | 6 months |
| 17 | Psychosis and affective disorder – difficult to engage | 6 months |
| 18 | Cognitive impairment (low need) | 6 months |
| 19 | Cognitive impairment or dementia (moderate need) | 6 months |
| 20 | Cognitive impairment or dementia (high need) | 6 months |
| 21 | Cognitive impairment (high physical need or engagement) | 6 months |

For example, if a patient was recorded as being in cluster 17 – psychosis and affective disorder, the timing for re-assessment would be within 6 months; therefore if the next re-assessment was completed after 9 months then this would not be within the required timing and should be recorded as 2.

If the persons first cluster diagnosis falls within the study data extraction period (with no previous clustering) then this person is classified as having been reassessed within the required timing (so enter 1 = yes)

**Q 26. Please tick all current major health morbidities recorded in the patient case notes.**

Please tick any major health morbidities recorded for the patient.

On eCR you may find this information within one of the assessments such as health and social needs assessments.

**Q 27. Please record all mental health medications within the data extraction dates and dose, including all oral and depot medications prescribed by mental health teams or by the practice listed on the medical record**

Please see appendix 1 for a list of medications and their generic names. Please use the codes provided to document the type of medication.

0 = None 3 = Bipolar disorder medication 6 = Other MH meds

1 = Antipsychotic (conventional) 4 = Anti-depressant medications

2 = Antipsychotic (Atypical) 5 = Anti-anxiety medications

Please record the date this drug was first prescribed if available and whether or not this prescription is ongoing i.e. is the patient still currently prescribed this medication at the end of the data extraction period.

For some patients it is difficult to determine exact date of prescription if they have been on a medication for some time. If the first prescription date is not available please estimate when the drug was first prescribed (or indicate when it was first recorded in patient notes)

It is OK to add the first of a particular month (e.g. “been on medication for 10 years” in notes - 01/01/2005). Please note if this has been an estimate.

**Q28. Has the patient had a blood pressure, bloods, BMI/waist measurement cholesterol or blood glucose levels checked and recorded in the two year data extraction period?**

**Please do not include physical health monitoring that has taken place while the patient was an inpatient on a ward.**

Please record any readings in the table provided, indicating the date they were performed.

If none of these metrics have been completed then please tick the box indicating this.

Please do not round the value up or down, please document the reading as is given on the system you are using. If a reading was abnormal (see Appendix 2 the UK Lester Adaptation Positive Cardio-metabolic Health Resource for ranges) then please record in the table whether or not the action as recommended by appendix 2 was taken and was recorded in the notes.

On eCR you may be able to search through the free text notes for key terms or look at the physical health modules for this information.

Please note, if the guidance has been followed as part of the physical health questionnaire that is completed every so often (e.g. annually) – so not necessarily on the dates that the reading has been taken, and not necessarily as a result of the reading as they were given this advice anyway then please record this as code 4 – recommended action taken as part of general physical health check. However if this action was in response to the reading then please code this as code 1 – yes recommended action taken as a result of abnormal reading.

**Q.29 Please complete the table to indicate the date of the last time each of the health checks was performed, or documented that the check has been performed. Please record ‘99’ if none have been documented in the notes.**

Here, we are interested in the relevant health checks that may have been performed, such as ECG and smear tests.

On eCR you may find this information on the rethink health and lifestyle module.

For some patients it is difficult to determine date of health checks. If there is some estimation of when the health check was performed then OK to add the first of a particular month (e.g. on 01/08/2014 documented that “last had ECG 3 years ago” – 01/08/2011)

Please record 00 if the records state that a particular health check has never been performed.

**Q 30. Is this patient a smoker?**

Please tick the appropriate box to indicate whether or not the patient is a smoker, or an ex-smoker. If the patient is a smoker please also record how many cigarettes are smoked per day, if this information is available.

**Q31. Has the patient had any help to stop smoking in the last 12 months e.g. referred to smoking cessation or prescribed nicotine replacement therapy?**

Please tick the appropriate box to indicate whether has been provided with any assistance to stop smoking, if this information is available.

On eCR you may find this information on the rethink health and lifestyle module.

**Q 32. Is there any evidence that this patient drank alcohol between the data extraction dates?**

Please tick the appropriate box to indicate whether or not the patient was recorded to have been drinking alcohol between the data extraction dates that we are interested in. If the patient does drink alcohol please also record how many units they drink per week, if this information is available.

If the amount of units is recorded in another frequency other than weeks then please record this in the free-text section provided.

**Q33. Is there any evidence that this patient was taking/has taken recreational or non-prescription drugs between the data extraction dates?**

Please tick the appropriate box to indicate whether or not the patient takes illegal drugs. If the patient does take illegal drugs please tick which of the drugs they have taken, if this information is available.

If the notes indicate that the patient has been taking drugs but you are not sure which category they fall into then please record this in the free text section provided.

On eCR you may find information about drug and alcohol use in the substance misuse section within the safety profiles, on the rethink health and lifestyle modules or within the free text.

**Q 34. Hospital admissions**

Please also include respite care in this question.

This section measures continuity of treatment across organisational boundaries—for example, through the transition from inpatient to outpatient services. In particular we need to know the date (dd/mm/yyyy) the patient was admitted (column 1) and discharged (column 2). If the patient is still in hospital indicate this by entering 88/88/88 in column 2.

This information may be found in one of the correspondence letters or may be available in the free text notes.

In column 3, please record the route of admission if known.

We also need to know the department/ specialty (box 2) the patient was admitted to.

- If the admission was for a mental health issue can you please indicate whether this was voluntary or compulsory. A compulsory admission would be when the patient has been formally detained under a section of the Mental Health Act 1983. A voluntary admission would be when the patient has an ‘informal’ status.
- Common private hospitals may include The Priory, St Andrews, Partnerships in care.

In the final column, we would like you to record the reason for admission.

**Q 35. Excluding inpatient admissions and mental health services has there been any referrals to hospital, A&E visits, walk in centre visits or out of hours contacts between the data extraction dates?**

This data may be included in the free text section of the notes, or may be found in one of the letters. This may require a bit of searching through the notes. Please do not include hospital inpatient admissions and mental health services in this question, as this data has been captured in other questions.

Within the table, please record in column 1 the type of contact using the codes in box 1.

Next, we would like you to indicate the speciality of the contact, using the codes in box 2.

In column 3 the date of referral (in the form dd/mm/yyyy) and in column 4 please indicate the reason for the contact using the codes provided.

In column 5 please record whether or not this was a single visit, and if it was not, please indicate the total number of visits for that particular referral in the sixth column.

**Q 36. Mental health service use**

Please complete the table regarding patient’s mental health service use between the data extraction dates. Please do not record mental health service use for dates during which the patient is an inpatient on a ward.

Please also use evidence contained in the free text in the medical notes, and attachments. We are hoping to capture fairly detailed information in this section so this may require quite a bit of searching through the records, or may require you to look at all contacts and notes documented for the patient.

We do not want you to capture information about inpatient admissions in this question, as this has already been captured in question 34. Therefore, please exclude any periods for which this patient was an inpatient on any kind of ward.

Ideally we would like you to collect information on both referrals and **all subsequent contacts** with the service if this is available. However, we realise that in some cases you may only be able to determine when the referrals were made to corresponding service.

Initially in the first column, we would like you to include the date of referral to the service. This is the original date (and may be before the data extraction period has begun) if unbroken service (ie they are still being seen by the service). If you do not know the referral date then please record 99. If this is a call to the crisis/support line then please record the date of the call in this referral box.

In the second column please fill in the date that the patient was seen by this service, or if this is a telephone call please record the date the call was made. The date of referral may be the same but the patient may have been seen numerous times by a particular service, therefore each contact with the service should be recorded on a separate line. We would like you to document all times that a service users has had contact with a professional from within a service.

In column 3 we would like you to record the service that the patient has been referred to or seen by. We have listed the main mental health services (see appendix 4 for more detailed notes on the services listed) but there may be others in which case include as other and specify the nature of the service. Furthermore mental health services may be configured so that the names we have used do not match the names of services in your area. Where relevant please use the most appropriate code and / or describe in more detail. Please also note that as we want to capture all mental health service use we also want to know about referrals made by other health and social care professionals.

Then in the 4^th^ column we’d like to know whether or not this was a self-referral, if this information is recorded.

In the next column, we’d like to know the method of contact, if this information is available. For example, whether this was over the phone, via a letter, face to face or another method.

The next column is to identify which professional the patient has actually seen.

It is also important for us to know whether or not the professional that has seen the patient is the care co-ordinator or not, please record this in column 7.

In the next column, (column 8) we are interested in the place that the contact took place, if this information is available, please use the codes provided to identify where the location was. If the professional visited or called but they did not speak to the service user then please record this as a ‘no access visit’. *If a patient is a permanent resident in supportive housing / residential home this is identified as ‘home visit’.*

Next, please record the date the patient was discharged from the service (if appropriate). If the patient has been discharged from the service but you do not know the date of discharge please record 99 in the date box. On the other hand, if the patient is still in receipt of the service please insert complete 88/88/8888. If this was a call to the out of hours crisis/support line then please record the date of the call in this column.

Finally, please record the reason for discharge using the codes provided.

**Q 36b**. **Within the CMHT (or equivalent) has there been any transfer between the ‘sub-teams’:**

For this question we would like you to record any transfer between internal teams and organisational changes that may have occurred. For example, if a patient was referred into the service when it was a CMHT and the team has since split into a CCTT and recovery team, and the patient has been transferred into one of the appropriate ‘sub team’s then we would like you to record this in this question.

Similarly a patient may have moved from a Community Mental Health Psychosis Team to a Psychosis and Recovery Team due to Trust organisational changes to local CMHTs rather than a move based on a patients reduction in symptoms or recovery.

**For questions 37-46, if there has been a period of time in which the patient has not been under the care of the CMHT then please record //. For example if the question asks how many psychiatrists the patient has seen within the first year of data extraction but the patient was only referred into the team within the second year of data collection then this can be recorded as // for the first year.**

**Q 37. Has the patient had a named care coordinator/case manager for any period during the data extraction period?**

This information may be contained in the case management section or in CPA documents, or a letter may have been sent to the patient to inform them of their case manager.

If the patient did have a named care coordinator for any period during the two year data extraction period that we are interested in answer question 38 next; if not skip to question 40.

**Q 38. Professional group of care coordinator**

There may be different names used for the care co-ordinator, for example case manager

Please complete the table to indicate which professional group this care coordinator belongs to using the codes in box 1 for each year of the data collection period. If there was a period during which there was no care co-ordinator, if so please use the code that specifies this.

**Q 39. Was the care coordinator the same for both time periods?**

If the patient has had a single named care coordinator for each of the two time periods please tell us whether or not this has been the same person.

We are asking this to measure relational or personal continuity (‘to provide one or more named individual professionals with whom the patient can establish and maintain a therapeutic relationship’) operationalised as evidence of a named person as the care coordinator.

**Q40. Has this patient had a named support worker/STR worker for any period during the data extraction dates?**

If yes, please continue to question 41. If no, please skip to question 44.

*Notes: Support workers from voluntary sector (for example residential homes) not to be recorded here but this could be noted in question 50 in which we are asking for an overview about the patient.*

**Q41**. **How many different named support workers/STR workers has this patient seen within the data extraction dates?**

For this first question, please record how many *different* support workers/STR workers the patient been allocated as their named support worker within the whole two year data extraction period.

**Q 42. How many different named support workers/STR workers has the patient seen in the first year of the data extraction period?**

Record the number of different named support workers/STR workers the patient has seen between in the **first** year of the data extraction dates.

**Q 43. How many named support workers/STR workers has the patient seen in the second year of the data extraction period?**

Record the number of different support workers/STR workers the patient has seen between in the **second** year of the data extraction dates.

**Q 44 . How many different psychiatrists has this patient seen within the data extraction dates?**

For this first question, please record how many *different* psychiatrists the patient has seen within the whole two year data extraction period.

For these questions it may be worth jotting down the names/initials of each appointment with the psychiatrist so that this question can be answered easily.

**Q 45. How many psychiatrists has the patient seen in the first year of the data extraction period?**

Record the number of different psychiatrists the patient has seen between in the **first** year of the data extraction dates. This does not include psychiatrists seen while the patient was an inpatient on a ward.

**Q 46. How many psychiatrists has the patient seen in the second year of the data extraction period?**

Record the number of different psychiatrists the patient has seen between in the **second** year of the data extraction dates. This does not include psychiatrists seen while the patient was an inpatient on a ward.

**Q.47. Has this patient been subject to a community treatment order within the data extraction period?**

Please indicate whether or not the patient has been under a community treatment order within the data extraction period?

**Q. 48 Has the patient been under section 117 within the data extraction period?**

For this question, please indicate whether or not the patient has been under a section 117 within the data extraction period?

**Q49. Have there been any CPA/review meetings held between the data extraction dates?**

If a patient is not on CPA reviews, they may instead have review meetings, if there have been either please complete the table. In this table we are interested in whether or not there is evidence that the CPA or review information has been provided to the service user, their carer and their GP; whether or not each of these actually attended the meeting and whether or not the individual has been involved in the development of their care plan.

**Q 50** Are there any other issues related to this patient's health care that you think are relevant which we have not been able to capture within this questionnaire? Please provide a short overview about the patient here.

In this question we would like you to add a summary paragraph about what you have read in the casenotes. Please see appendix 5 – Qualitative summary guidance document for more details.

**Q51. If there were questions/sections you found difficult to complete for this patient, please tell us which question/section and why?**

As we’ve expressed throughout the manual this type of data collection is not easy. We would like you to indicate the level of confidence you have in the data collected and indicate any sections which were difficult (if any) to complete for each patient.

**Finally**

• Please check for missing data - are there any blank spaces? Or did you intend to go back and check the records for any items?

• If possible we would like to have all the forms returned to us no later than date.

The data may take some months to process. When the data has been analysed, findings will be reported in the form of journal articles. You may however hear from us sooner if we have any queries related to your data.

***Thank you very much for participating in the study.***

***We realise this type of data collection is arduous and appreciate your efforts to collect good quality data.***

***We are sure that the results will be useful.***

If you have any queries about the study or about filling in the forms please use the contact details on the front page.

**Appendix 1 - Alphabetical List of Medications by Generic Name Generic Name Trade Name**

**1) Antipsychotic Medications (conventional)**

Chlorpromazine (conventional) Largactil

Fluphenazine (conventional) Modecate

Haloperidol (conventional) Haldol

Perphenazine (conventional) Fentazin

Pimozide (conventional) Orap

Sulpiride (conventional) Dolmatil

Trifluoperazine (conventional) Stelazine

**2) Antipsychotic Medications (atypical)**

Amisulpiride (atypical) Solian

Aripiprazole (atypical) Abilify

Clozapine (atypical) Clozaril

Olanzapine (atypical) Zyprexa

Quetiapine (atypical) Seroquel

Risperidone (atypical) Risperdal

**3) Bipolar disorder Medications**

Carbamazepine Tegretol

Gabapentin Neurontin

Lamotrigine Lamictal

Lithium carbonate Priadel

Lithium citrate Priadel

Valproic acid Depakote

topimarate Topamax

**4) Antidepressant Medications**

Amitriptyline (tricylcic) Elavil

Citalopram (SSRI) Cipramil

Clomipramine (tricylcic) Anafranil

Dosulepin (tricylcic) dothiepin

Doxepin (tricylcic) Sinepin

Escitalopram (SSRI) Cipralex

Fluvoxamine (SSRI) Faverin

Fluoxetine (SSRI) Prozac

Imipramine (tricylcic) Tofranil

Isocarboxazid (MAOI) Isocarboxazid

Lofepramine (tricyclic) Lofepramine

Mianserin (tricyclic) Mianserin

Mirtazapine

(presynaptic alpha2-adrenoreceptor antagonist)

Zispin

Nortriptyline (tricylcic) Allegron

Paroxetine (SSRI) Seroxat

Phenelzine (MAOI) Nardil

Reboxitine

(selective inhibitor of noradrenaline re-uptake)

Edronax

Sertraline (SSRI) Lustral

Tranylcypromine (MAOI) Tranylcypromine

Trazodone (SSRI) Molipaxin

Trimipramine (tricylcic) Surmontil

Venlafaxine (SNRI) Effexor

**5) Antianxiety Medications**

Alprazolam (benzodiazepines) Xanax

Buspirone Buspar

Chlordiazepoxide (benzodiazepines) Librium

Clonazepam (benzodiazepines) Rivotril

Diazepam (benzodiazepines) Valium

Lorazepam (benzodiazepines) Ativan

Oxazepam (benzodiazepines) Oxazepam

**Appendix 2**

In addition – Waist circumference would count as an alternative to BMI, please use the following guidance (from NHS Choices website) if waist circumference has been recorded:

You have a higher risk of health problems if your waist size is:

more than 94cm (37 inches) if you're a man more than 80cm (31.5 inches) if you're a woman Your risk of health problems is even higher if your waist size is:

more than 102cm (40 inches) if you're a man more than 88cm (34.5 inches) if you're a woman

**Appendix 3 – Practice codes**

**Site**

| 1 |  |
| --- | --- |
| 2 |  |
| 3 |  |
| 4 |  |
| 5 |  |
| 6 |  |
| 7 |  |
| 8 |  |
| 9 |  |
| 10 |  |
| 11 |  |
| 12 |  |
| 13 |  |
| 14 |  |
| 15 |  |
| 16 |  |
| 17 |  |
| 18 |  |
| 19 |  |
| 20 |  |
| 21 |  |
| 22 |  |
|  |  |

Site

| **GP Practice Number** | **GP Practice Name** |
| --- | --- |
| 1 |  |
| 2 |  |
| 3 |  |
| 4 |  |
| 5 |  |
| 6 |  |
| 7 |  |
| 8 |  |
| 9 |  |
| 10 |  |
| 11 |  |
| 12 |  |
| 13 |  |
| 14 |  |
| 15 |  |
| 16 |  |
| 17 |  |
| 18 |  |
| 19 |  |
| 20 |  |
| 21 |  |
| 22 |  |
| 23 |  |
| 24 |  |
| 25 |  |
| 26 |  |
| 27 |  |
| 28 |  |
| 29 |  |
| 30 |  |

Add in codes of GP practices for other localities.

**Appendix 4 - Information about the services for question 36**

**1 CMHT (or equivalent) – general**

Community mental health teams (CMHTs) are multidisciplinary, multi-agency teams offering specialist assessment, treatment and care to adults with mental health problems, both in their own homes and in the community.

This code would include a general contact (scheduled or unscheduled) by the community mental health team (including the CCTT or recovery team in areas whereby they have been divided into these separate teams). This may include a visit or phone call to see how the patient is feeling, a meeting to discuss their medications or an unscheduled contact.

**For codes 1-4 in Lancashire, both the CCTT and the recovery team would come under this code. Any transfer between the teams would be captured on question 36b.**

**2 CMHT (or equivalent) - depot clinic**

Community mental health teams (CMHTs) are multidisciplinary, multi-agency teams offering specialist assessment, treatment and care to adults with mental health problems, both in their own homes and in the community.

This code would include a contact when the patient has attended a clinic to receive their depot injection by the community mental health team (or equivalent). We have included this code because we would like to differentiate between ‘therapeutic encounters’ as in the code above and when the patient has attended for a clinic simply to receive their depot medication but has not received a therapeutic encounter with a member of the team.

**3 CMHT (or equivalent) – Home depot**

Community mental health teams (CMHTs) are multidisciplinary, multi-agency teams offering specialist assessment, treatment and care to adults with mental health problems, both in their own homes and in the community.

We have included this code to capture when a patient is seen at home to administer their depot injection.

**4 CMHT (or equivalent) - medication clinic**

Community mental health teams (CMHTs) are multidisciplinary, multi-agency teams offering specialist assessment, treatment and care to adults with mental health problems, both in their own homes and in the community

This code would include a contact when the patient has attended a medication clinic (for example a clozapine clinic) with the community mental health team (or equivalent). We have included this code because we would like to differentiate between ‘therapeutic encounters’ as in the code above and when the patient has attended for a clinic simply to receive their have their bloods taken/physical health checked and has not received a therapeutic encounter with a member of the team.

**5 Outpatient psychiatry**

Please use this code if the patient has been referred to or seen by any outpatient psychiatry **excluding** from the CMHT which will be coded as CMHT or equivalent.

**6 Home treatment teams/crisis resolution**

Crisis resolution and home treatment teams (CRHTs) treat people with severe mental health conditions who are currently experiencing an acute and severe psychiatric crisis that, without the involvement of the CRHT, would require hospitalisation. Because of the nature of their work, CRHTs offer a 24-hour service, and cases are often referred to them through accident and emergency (A&E) departments or the police service.

CRHTs will usually review a person in their own home, but they may also see people in other community settings, such as dedicated crisis houses or a day centres.

**7 Day care**

Please use this code if the service user has been referred to or seen by any type of day services or day centres.

**8 Rehabilitation/recovery**

Rehabilitation and recovery teams are multidisciplinary teams that focus on the needs of people with long-term severe mental illness. The teams provide ongoing care and support, particularly focused on:

- maintaining health
- preventing relapses
- developing social networks
- establishing meaningful activities in people's lives

These teams are not available in all areas, but if you do find that the patient has been in contact with this service then please use this code.

**9 Assertive community treatment**

Assertive outreach teams (AOTs) are community treatment teams that look after adults with severe mental health conditions and personality disorders. These people may find it difficult to work with services, have been admitted to hospital a number of times, and may have other problems such as violence, self-harm, homelessness or substance abuse.

AOTs provide a patient-centred approach, offering an intensive and long-term relationship that builds trust. This is so they can help identify a person's needs, career aspirations and strengths. They also support families and carers where necessary.

**10 Social services**

We understand that this is a very broad category. Therefore, if there is more detail about which branch of social services that the patient has been seen by/referred to then please specify this in the next column.

**11 Forensic**

Forensic mental health services work with people who have mental health conditions and have committed a serious criminal offence, or are thought to be at high risk of committing an offence. Forensic mental health services may care for people in secure hospitals or prisons. Most of the people who are in need of such services are thought to be a risk to both themselves and others. Community forensic mental health services can also care for people out in the community following discharge from a secure hospital or prison.

**12 Back to primary care**

Please use this code if the patient has been referred back to primary care, for example back to their GP practice.

**13 Early Intervention Services**

Early intervention for psychosis teams (EIPTs) work with people between the ages of 18 and 35 who may be experiencing their first episode of psychosis. In some areas, they work with people who are younger than 18.

**14 CBT**

Please use this code when the service user has been referred to or see by Cognitive behavioural therapies. This could be in the form of individual therapy, group therapy or perhaps a computerised cognitive behavioural therapy.

**15 Family therapy**

Please use this code if the service user has been referred to or seen by family therapy services.

**16 Other types of psychotherapy**

Please use this code for when the service user has seen or been referred to any other type of psychotherapy services (other than family therapy or CBT which we have included as separate codes). Other forms of psychotherapy may include:

- Psychodynamic (psychoanalytic)
- Cognitive analytical therapy (CAT)
- Interpersonal psychotherapy (IPT)
- Humanistic therapies

**17 Crisis Housing**

Crisis houses offer safe short-term accommodation and support to people experiencing a mental health crisis. They are used when home treatment is not suitable, or as a short-term alternative to hospital admission.

Some crisis houses accept self-referrals, but often referrals are taken from secondary mental health services.

**18 Voluntary organisation support service**

Please use this code for when the service user has been referred to or seen by one of the voluntary organisation support services.

**19 Psychologist**

Please use this code if the service user has been referred to or seen by psychology services.

**20 Drugs and alcohol services**

Please use this code for when the service user has been referred to or seen by one of the drugs or alcohol services.

**21 Homelessness services**

Please use this code for when the service user has been referred to or seen by homelessness services.

**22 IAPT**

IAPT is Improving Access to Psychological Therapies, please use this code if the service user has been referred to IAPT services.

**23 Counselling**

Please use this code if the service user has been seen by or referred to counselling.

**24 Chaplaincy**

Please use this code if the service user has been referred to or seen by chaplaincy.

**25 Art therapy**

Please use this code if the service user has been seen by or referred to art therapy services.

**26 Support Groups**

Please use this code if the service user has been referred to or seen by additional support groups for example Bipolar UK.

**27 Other/unsure which category (please specify)**

Please use this code when the service user has seen one or been referred to one of the mental health services, but the service is either not included in our lists of codes or you are unsure which category the service falls into. If this is the case, please specify the name of the service as detailed in the notes.

**99 Not recorded**

Please use this code for when a contact has been made, but it is not recorded which service the professional is from.

If the service is social services, we realise that this covers a wide range of services and so we have included an additional column in which we would like you to record the specific branch of social services.

If the patient has been referred to a service but you are unsure which of the categories provided that this comes under then please use column three to specify the name of the service.

**Appendix 5 - Qualitative Summary Guidance:**

Please complete a short summary/overview about the patient. Please also use this space to add any important points that may not necessarily be explicitly captured within the data extraction tool, or to add any explanation to the data that you feel may be useful. Important points to note may include:

**Basic information:**

- **Age & gender**

**Diagnosis:**

- **Diagnosis information:** e.g. *“long history of schizophrenia since age 25”*

**Referral information:**

- **Referral information** e.g. *“Referred to the CMHT by GP via single point of access team.”*

**Summary of team involvement:**

- **CPN/social worker/STR/other team member support** - where possible notes about what this linked to for example finances, housing, community activities e.g. *“requesting medication review therefore main involvement with the team has been with CPN with oversight from the psychiatrist”* or *“extensive support from social worker due to issues with benefits”*
- **Involvement from other services** e.g. “*receiving support from CAB in relation to finances*”

**Social support/lifestyle:**

- **Comments about social links/isolation** e.g. *“reports that she has a good social network including daughter, friends and the local church group”*
- **Key information about lifestyle** e.g. “*lived with mother for 10 years*”

**Medication:**

- **Medication/non-adherence/shared decision making e.g. “***patient has been* *Involved in discussions about changes to medication*” or “*medications prescribed by GP*” or *“relapse in mental health when stopped collecting his prescribed medication from the GP practice”*

**Care planning:**

- **Comments about care planning** e.g. “*Comprehensive care plan produced but primary care not included in preparing it*”

**Other:**

- **Any information about admissions** e.g. *“Last admission was voluntary admission in Spring 2009 for 3 weeks, no further admissions since this date”*
- **Other key points that have not been captured in the tool** “*absconded from hospital*” or “*has key support worker in supportive housing*”
- **Reason for any gaps in data** e.g. “*limited data available as patient did not want to engage with team*”

**Discharge (if applicable):**

- **Reason for discharge** e.g. “*discharged back to primary care at patient’s request as did not want to engage with the team*”

**Examples:**

- *‘Male, aged 47, lives in supportive/residential home. Extensive key worker support here. Long term patient – diagnosed with schizophrenia 25 years ago. Lived with mother up until 10 years ago. No life outside of residential home. No work. Socially isolated. Acutely ill in past with previous admission. Symptoms managed with medication’.*
- *“Male, aged 42, socially isolative. Lives with mother and acts as carer for her physical conditions, which is a stressor for him. Diagnosis of schizophrenia but currently in remission, has been stable for many years. Sees the team mainly for depot injection. Physical health monitored by GP. Aware of signs of relapse and aware of what to do in these circumstances.”*
- *“Female, aged 45, several referrals to the CCTT in the past 2 years, from self and from others. Accepted into team then frequently disengages and fails to attend appointments, therefore limited data available. Treated for depression for past three years, query bipolar disorder. Lives with spouse, is employed but currently on sick leave.”*
