# Supplementary material for: Status of primary and secondary mental healthcare of people with severe mental illness: an epidemiological study from the UK PARTNERS2 programme
Source: BJPsych Open. 2021 Feb 15;7(2):e53. doi: 10.1192/bjo.2021.10 (PMC8058911; doi:10.1192/bjo.2021.10)
Supplement: Supplementary file 1 [file S2056472421000107sup001.zip › ONLINE_DET_Primary_Care.pdf]

# PARTNERS2: ASSESSMENT OF LOCAL HEALTH CARE PATHWAYS AND CURRENT SERVICES FOR PEOPLE WITH SEVERE MENTAL ILLNESS (Primary Care)

*PATIENT DATA EXTRACTION FORM TO BE COMPLETED USING THE STUDY MANUAL*

## A: Your details

**1.** Name of CSO:.......................................................................................................................

## B: Patient ID and date of data extraction

**2**. Unique patient study number: ☐☐☐☐ **3**. GP practice Code ☐ See Manual for codes

Site number:
1 = Birmingham
2 = Devon
3 = Lancashire

**4.** Date of data extraction: ☐☐ / ☐☐ / ☐☐☐☐ **5**. Site number: ☐

**6.** Dates of data period (dd/mm/yyyy):

From ☐☐ / ☐☐ / ☐☐☐☐ to ☐☐ / ☐☐ / ☐☐☐☐

**7**. GP practice postcode: ☐☐ ☐☐ ☐☐☐

## C: Patient demographic details

**8**. Patient age (in years) at August 2014: ☐☐☐

**9**. Patient gender: ☐ 1 Male ☐ 2 Female ☐ 3 Transgender

**10**. Is the patient British? ☐ 1 Yes ☐ 2 No ☐ 99 Not recorded

**11**. Patient ethnicity ☐

White
1 = White British
2 = White Irish
3 = Gypsy or Irish traveler
4 = White other

Mixed/multiple ethnic groups
5 = White and black Caribbean
6 = White and black African
7 = White and black Asian
8 = Any other mixed/multiple

Asian/Asian British
9 = Indian
10 = Pakistani
11 = Bangladeshi
12 = Chinese
13 = Any other Asian background

Black/African/Caribbean/Black British
14 = African
15 = Caribbean
16 = Any other black/African/Caribbean background

Other Ethnic Groups
17 = Arab
18 = Any other ethnic group - please specify…………
99 = Not recorded

**12**. Usual living situation: ☐

1 = Alone
2 = With parents
3 = With spouse/partner
4 = With spouse + children
5 = With children (lone parent)
6 = With other family
7 = With non-family
8 = Group home
9 = Other - please specify ……………….
99 = Not recorded

Please include respite on question 34

**13**. Does the patient have any children? If yes, please specify the number of children within each age range.

☐ 1 Yes ☐ 2 No ☐ 99 Not recorded

☐ 1 Under 18 ☐ 2 Over 18 ☐ 99 Not recorded

**14**. Has this patient been homeless in the last 5 years?

☐ 1 Yes ☐ 2 No ☐ 99 Not recorded

**15**. Current employment: ☐

1 = In full-time employment
2 = In part-time employment
3 = Self employed
4 = Not working – housewife/househusband
5 = Not working – retired
6 = Not working – unemployed
7 = Not working – other…………………..….
8 = Voluntary work
9 = Full time carer
10 = Other (please specify):

………..………………………………..
99 = Not recorded

**16**. Does this patient **currently** receive any benefits (including permitted work, personal independence payments, employment support allowance, or disability allowance)?

☐ 1 Yes ☐ 2 No ☐ 99 Not recorded

**17**. Refugee status

☐ 1 Yes ☐ 2 No ☐ 99 Not recorded

**18**. Asylum seeker status

☐ 1 Yes ☐ 2 No ☐ 99 Not recorded

## D: Patient health and medication details

**19**. What is this patient's current (most recent) serious mental illness diagnosis? ☐

| | |
|---|---|
| 0 = Not recorded<br>1 = Schizophrenia Eu20<br>2 = Schizotypal personality Eu21<br>3 = Persistent delusional Disorder Eu22<br>4 = Acute/Transient psychotic disorders Eu23<br>5 = Induced delusional disorder Eu24<br>6 = Schizoaffective disorders Eu25<br>7 = Manic Episodes Eu30<br>8 = Bipolar Disorder Eu31<br>9 = Severe Depression with Psychosis Eu323<br>10 = Schizotypal Personality E2122 | 11 = Other (Please specify):<br>………………………………………………<br>99 = Not recorded |

**20**. Please specify the exact diagnosis if recorded

…………………………………………………………..........

**21.** What date was this diagnosis first made? ☐☐ / ☐☐ / ☐☐ ☐☐ or tick ☐ for not recorded

Is this date exact or an estimate? 1 Exact ☐ 2 Estimate ☐

**22**. What date was this patient added to the SMI register? ☐☐ / ☐☐ / ☐☐ ☐☐

**23**. Who made the diagnosis? ☐ 1 = GP 2 = Psychiatrist 3 = Other (please specify) …………………………………………… 99 = Not Recorded

**24**. Is there evidence of a dual diagnosis (i.e. a substance misuse or alcohol problem) for this patient?

☐ 1 Yes ☐ 2 No ☐ 99 Not recorded

**25**. Please tick **any** major health morbidities recorded for this patient**:**

| | | |
|---|---|---|
| ☐ 1 Diabetes | ☐ 8 Ischaemic heart disease | ☐ 15 Cancer |
| ☐ 2 Asthma | ☐ 9 Heart failure | ☐ 16 Osteoarthritis |
| ☐ 3 COPD | ☐ 10 Chronic kidney disease | ☐ 17 Obesity |
| ☐ 4 Epilepsy | ☐ 11 Depression | ☐ 18 Visual problems |
| ☐ 5 Hypertension | ☐ 12 Learning disability | ☐ 19 None |
| ☐ 6 Stroke | ☐ 13 Hearing problems | ☐ 20 Other (please specify) |
| ☐ 7 Thyroid disorder | ☐ 14 Rheumatoid arthritis | …………………………………….. |

……………………………………..

……………………………………..

…………………………………….

**26**. Please record all current mental health medications (use the codes in box 1) listed on the medical record (see Appendix 1 of the instruction manual for a list of medications by generic name)

**Box 1: Medication type**

1 = Antipsychotic (conventional)
2 = Antipsychotic (atypical)
3 = Bipolar disorder medications
4 = Antidepressant medications
5 = Antianxiety medications
6 = Other mental health meds
99 – Data not recorded/not known/missing
0 = No prescription

| Medication type (Box 1) | Name of medication | Date of prescription dd/mm/yyyy | Ongoing?<br>1 = Yes<br>2 = No<br>99 = Data not recorded/not known/missing |
|---|---|---|---|
| | | | |
| | | | |
| | | | |
| | | | |
| | | | |
| | | | |
| | | | |
| | | | |
| | | | |
| | | | |
| | | | |
| | | | |
| | | | |
| | | | |
| | | | |
| | | | |
| | | | |

Please continue on additional sheets if necessary

**27**.

Has the patient had a blood pressure, bloods, BMI, cholesterol or blood glucose levels checked and recorded between data extraction dates?

☐ 1 Yes ☐ 2 No ☐ 99 Not recorded

Box 1 (See appendix in manual -UK Lester Tool for information about action)
1 = Yes recommended action taken
2 = No action taken
3 = N/A - results were in range
99 = Not recorded

If yes, please record in the tables below.
Please complete all recorded for the data extraction period.
Please continue on additional sheets if necessary.

| **Date Recorded Dd/mm/yyyy** | Please document BP (systolic/ diastolic) or record 99 if not taken | If abnormal was the recommended action taken and documented in the notes (Box 1) |
|---|---|---|
| | | |
| | | |
| | | |
| | | |
| | | |
| | | |
| | | |
| | | |

| **Date Recorded Dd/mm/yyyy** | Please document BMI or record 99 if not taken | If abnormal was the recommended action taken and documented in the notes (Box 1 ) |
|---|---|---|
| | | |
| | | |
| | | |
| | | |
| | | |
| | | |
| | | |

| **Date Recorded Dd/mm/yyyy** | Please document cholesterol or record 99 if not recorded | If abnormal was the recommended action taken and documented in the notes (Box 1 ) |
|---|---|---|
| | | |
| | | |
| | | |
| | | |
| | | |
| | | |

| Date Recorded Dd/mm/yyyy | Please document HbA1c and glucose levels or record 99 if not taken | If abnormal was the recommended action taken and documented in the notes (Box 1 ) |
|---|---|---|
| | | |
| | | |
| | | |
| | | |
| | | |
| | | |

**28.** Please fill in the following table for the most recent date that the health checks were performed, or record 99 in date column if not recorded.

1 = Offered health checks but not performed
// = Not applicable
99 = Not recorded

| Please record the most recent date that the following health checks were recorded | Date (dd/mm/yyyy) |
|---|---|
| ECG | |
| Cervical smear (females only) | |
| Mammogram (females over 50 only) | |

## : Lifestyle

**29**. Is this patient a smoker?

☐ 1 Yes ☐ 2 No ☐ 3 Ex- smoker ☐ 99 Not recorded

☐ 1 1-10 ☐ 2 11-20 ☐ 3 21-30 ☐ 4 30+ per day ☐ 99 Not recorded

If yes, please complete question 30. If not, skip to question 31.

**30.** Has the patient had any help to stop smoking between the data extraction dates? e.g. referred to smoking cessation or prescribed nicotine replacement therapy?

☐ 1 Yes ☐ 2 No ☐ 3Patient declined ☐ 99 Not recorded

**31**. Is there any evidence that this patient drank alcohol between the data extraction dates?

☐ 1 Yes ☐ 2 No ☐ 99 Not recorded

If yes, please document the most recent level of alcohol consumption **per week** as documented in the notes.

☐ 1 1-5 units ☐ 2 6-10 units ☐ 3 11-15 units

☐ 4 16-20 units ☐ 5 21-25 units ☐ 6 26-30 units

☐ 7 31-35units ☐ 8 >35 units

☐ 9 Other amount/other frequency (please specify)……………………………………………………

☐ 99 Not recorded

**32.** Is there any evidence that this patient took recreational or non-prescription drugs between the data extraction dates?

☐ 1 Yes ☐ 2 No ☐ 99 Not recorded

☐ 1Ecstasy ☐ 2 LSD ☐ 3 Heroin

☐ 4Cocaine ☐ 5Crack Cocaine ☐ 6Magic Mushrooms

☐ 7Methadone ☐ 8Meth Amphetamine (Crystal Meth)

☐ 9Amphetamines ☐ 10Cannabis ☐ 11Codeine

☐ 12Methylphenidate ☐ 13Pholcodine ☐ 14 Other – Please specify:

☐ 99 Not recorded

………………………………………………………..

………………………………………………………..

………………………………………………………..

……………………………………………………..

## F: Patient health service use

**33**. Have there been any contacts with **primary health care staff from the practice** within the data extraction dates?

☐ 1Yes ☐ 2No ☐ 99Not Recorded

**Box 1: Type of contact**

1 = With GP
2 = With practice nurse/nurse clinician/nurse practitioner
3 = With primary care mental health worker
4 = Health care assistant
5 = Other – please specify
99 = Data not known/missing/unsure

**Box 2: Place of contact**

1 = Practice
2 = Home
3 = Telephone
4 = Third Party telephone liaison about the patient (incoming)
5 = Third party telephone liaison about the patient (outgoing)
6 = Other - please specify
99 = Data not know/missing/unsure

**Box 3: Reason for contact**

1 = New physical health – symptoms, diagnosis and referrals

2 = Physical health or ongoing physical existing conditions

3 = Health promoting activities

4 = Social, family, housing, employment, financial

5 = New mental health conditions including physical effect as a result of mental health (e.g. self harm)

6 = Mental health or ongoing mental health existing conditions including physical effects as a result of mental health (e.g. self harm)

7 = Other – please specify

| Date of contact (dd/mm/yyyy) | Type of contact (Box1) | Initials of staff member | Place of contact (Box 2) | Reason for contact (Box 3) |
|---|---|---|---|---|
| | | | | |
| | | | | |
| | | | | |
| | | | | |
| | | | | |
| | | | | |
| | | | | |
| | | | | |
| | | | | |
| | | | | |
| | | | | |
| | | | | |

| | | | | |
|---|---|---|---|---|
| | | | | |
| | | | | |
| | | | | |
| | | | | |
| | | | | |
| | | | | |
| | | | | |
| | | | | |
| | | | | |
| | | | | |
| | | | | |
| | | | | |
| | | | | |
| | | | | |
| | | | | |
| | | | | |
| | | | | |
| | | | | |
| | | | | |
| | | | | |
| | | | | |
| | | | | |
| | | | | |
| | | | | |
| | | | | |
| | | | | |
| | | | | |
| | | | | |
| | | | | |

Please continue on additional sheets if necessary

**34**. Have there been any **hospital admissions** between the data extraction dates?

☐ 1Yes ☐ 2No ☐ 99Not Recorded

If yes, please complete the table below

**Box 1: Route of admission**
1 = A&E
2 = GP
3 = Crisis team
4 = Other (please specify)
99 = Not recorded/not known

**Box 2: Specialty**
1 = Mental health (voluntary)
2 = Mental health (compulsory)
3 = Cardiology
4 = Respiratory
5 = Ophthalmology
6 = ENT
7 = Gastroenterology
8 = Neurology
9 = Orthopedics
10 = Physiotherapy
11 = Cancer care
12 = Palliative care
13 = Other surgery
14 = Dermatology
15 = Stroke
16 = Gynecology
17 = Chiropody
18 = Other (please specify)
19 = Private hospital
20 = Respite care
99 = Not recorded/not known

**Box 3 Reason for admission**
1 = Suicide attempt / overdose
2 = Self harm
3 = Alcohol / substance abuse
4 = Accidental injury
5 = Request for psychiatric help
6 = Physical health problem
7 = Prescription run out
8 = Diagnosis
9 = Specialist investigation
10 = Physiotherapy
11 = Antenatal care
12= Other/unsure which category (please specify)
99 = Not recorded

| **Date admitted (dd/mm/yyyy)** | **Route of admission (Box 1)** | **Date discharged (dd/mm/yyyy)**<br><br>**If patient is still in hospital indicate by entering 88/88/88** | **Specialty (Box 2)** | **Reason for admission (Box 3)** |
|---|---|---|---|---|
| | | | | |
| | | | | |
| | | | | |
| | | | | |
| | | | | |
| | | | | |
| | | | | |

| Date admitted (dd/mm/yyyy) | Route of admission (Box 1) | Date discharged (dd/mm/yyyy)<br><br>If patient is still in hospital indicate by entering 88/88/88 | Specialty (Box 2) | Reason for admission<br>(Box 3) |
|---|---|---|---|---|
| | | | | |
| | | | | |
| | | | | |
| | | | | |
| | | | | |
| | | | | |
| | | | | |
| | | | | |
| | | | | |
| | | | | |
| | | | | |
| | | | | |
| | | | | |
| | | | | |
| | | | | |
| | | | | |
| | | | | |
| | | | | |
| | | | | |
| | | | | |

Please continue on additional sheets if necessary

**35**. Excluding inpatient admissions and mental health services, have there been any referrals to hospital, A&E visits, Walk in Centre visits or out of hours contacts between the data extraction dates?

Yes ☐ No ☐ Not Recorded ☐

**Type of contact (Box 1)**

1 = Referrals to hospital outpatient/clinic services (not inpatient admission)
2 = A&E visits
3 = Walk-in-Centre visits
4 = Out of hours primary care contact
5 = Pharmacy
6 = Called Ambulance
7 = Other (please specify in table)
99 = Data not recorded/not known/missing

**Box 2: Specialty**

1 = Cardiology
2 = Respiratory
3 = Ophthalmology
4 = ENT
5 = Gastroenterology
6 = Neurology
7 = Orthopedics
8 = Physiotherapy
9 = Cancer care
10 = Palliative care
11 = Other surgery
12 = Dermatology
13 = Stroke
14 = Gynecology
15 = Chiropody
16 = Other (please specify)
99 = Not recorded/not known

**Box 3 Reason for contact**

1 = Suicide attempt / overdose
2 = Self harm
3 = Alcohol / substance abuse
4 = Accidental injury
5 = Request for psychiatric help
6 = Physical health problem
7 = Prescription run out
8 = Diagnosis
9 = Specialist investigation
10 = Physiotherapy
11 = Antenatal care
12= Other (please specify in table)
99 = Data not recorded/not known/missing

| **Date of referral (dd/mm/yyyy)** | **Type (Box 1)** | **Specialty (Box 2)** | **Reason for referral (Box 3)** | **Single visit (yes/no)** | **If more than 1 visit per referral, please give total number of visits** |
|---|---|---|---|---|---|
| | | | | | |
| | | | | | |
| | | | | | |
| | | | | | |
| | | | | | |
| | | | | | |
| | | | | | |
| | | | | | |
| | | | | | |

| | | | | | |
|---|---|---|---|---|---|
| | | | | | |
| | | | | | |
| | | | | | |
| | | | | | |
| | | | | | |
| | | | | | |
| | | | | | |
| | | | | | |
| | | | | | |
| | | | | | |
| | | | | | |
| | | | | | |
| | | | | | |
| | | | | | |
| | | | | | |
| | | | | | |
| | | | | | |
| | | | | | |
| | | | | | |
| | | | | | |
| | | | | | |
| | | | | | |

Please continue on additional sheets if necessary

**36**. Excluding inpatient admissions, please complete the following table regarding the patient's contact with mental health services between the *data extraction* dates?
Or if not recorded please tick: ☐
(see manual)

**Box 1: Mental health service referred to:**

1 = Community mental health team(or equivalent)
2 = Outpatient psychiatry
3 = Home treatment teams / crisis resolution
4 = Day care
5 = Rehabilitation / recovery
6 = Assertive community treatment
7 = Social services (please specify type)
8 = Forensic
9 = Back to primary care
10 = Early intervention services
11 = CBT
12 = Family therapy
13 = Other or unsure which category (please specify)
99 = not recorded

**Box 2: Was the patient seen by the service referred to?**

1 = Yes and referral letter present
2 = Yes but no documentation received
3 = No, patient not seen
99 = Not recorded/ not known

**Box 3: If discharged - why was this?**

1 = DNA
2 = lost to follow up for no apparent reason
3 = Patient request
4 = Team felt no further contact needed
5 = Other (please specify)
6 = Inappropriate referral
99 = not recorded

| Service? (Box 1) | If Box 1 = 7, please specify type of social services | If Box 1= 13, please specify | Date referred (dd/mm/yyyy) (referral may be prior to data extraction dates) | Seen? (Box 2) | Date discharged (dd/mm/yyyy) | Discharge reason? (Box 3) | Number of documents received from service/ team within this period |
|---|---|---|---|---|---|---|---|
| ……… | | | / / | ☐ | / / | …………… | |
| ……… | | | / / | ☐ | / / | …………… | |
| ……… | | | / / | ☐ | / / | …………… | |
| ……… | | | / / | ☐ | / / | …………… | |
| ……… | | | / / | ☐ | / / | …………… | |

| Service? (Box 1) | If Box 1 = 7, please specify type of social services | If Box 1= 13, please specify | Date referred (dd/mm/yyyy) (referral may be prior to data extraction dates) | Seen? (Box 2) | Date discharged (dd/mm/yyyy) | Discharge reason? (Box 3) | Number of documents received from service/ team within this period |
|---|---|---|---|---|---|---|---|
| ……… | | | / / | ☐ | / / | ………….. | |
| ……… | | | / / | ☐ | / / | ………….. | |
| ……… | | | / / | ☐ | / / | ………….. | |
| ……… | | | / / | ☐ | / / | ………….. | |
| ……… | | | / / | ☐ | / / | ………….. | |
| ……… | | | / / | ☐ | / / | ………….. | |
| ……… | | | / / | ☐ | / / | ………….. | |
| ……… | | | / / | ☐ | / / | ………….. | |
| ……… | | | / / | ☐ | / / | ………….. | |
| ……… | | | / / | ☐ | / / | ………….. | |
| ……… | | | / / | ☐ | / / | ………….. | |
| ……… | | | / / | ☐ | / / | ………….. | |
| ……… | | | / / | ☐ | / / | ………….. | |
| ……… | | | / / | ☐ | / / | ………….. | |
| ……… | | | / / | ☐ | / / | ………….. | |

**37**. Has this person had a named care co-coordinator/case manager at any period during **the data collection period**?

- ☐ 1 Yes → Go to question 38
- ☐ 2 No → Go to question 40
- ☐ 99 Not recorded → Go to question 40

| **Box 1 Care coordinators/case managers professional group** | |
|---|---|
| 1 = Social worker | 8 = Staff grade psychiatrist |
| 2 = CPN | 9 = Mental Health Practitioner |
| 3 = Occupational therapist | 10 = Recovery Practitioner |
| 4 = Clinical psychologist | 11 = Other or unsure which category (please specify in table) |
| 5 = Consultant psychiatrist | 12 = Period with no care coordinator/case manager |
| 6 = Mental health worker in secondary care | 99 = Not recorded |
| 7 = Junior doctor | |

**38**. Please specify the professional groups and number of care coordinators/case managers? – use codes from **Box 1**.

| Year 1 | | Year 2 | |
|---|---|---|---|
| Professional Group | Total number form this professional group | Professional Group | Total number form this professional group |
| | | | |
| | | | |
| | | | |
| | | | |
| | | | |
| | | | |
| | | | |
| | | | |
| | | | |

**39.** If there has been a single named care coordinator/case manager for both time periods, was the care coordinator the same person for each time period?

☐ 1 Yes ☐ 2 No ☐ 99 Not recorded

**40**. How many different psychiatrists has this patient seen within **the 2 year data extraction period?**

☐☐

**41**. How many different psychiatrists (any type of psychiatrist, e.g. junior) has the service user seen in the first year of the data extraction period**?**

☐☐

**42**. How many different psychiatrists has the service user seen in the second year of the data extraction period**?**

☐☐

**We strongly advise that you refer back to the manual and information recorded before completing the next question**

**43.** Thinking about the mental health care this patient has received for the whole two year time period under consideration please tick each statement that applies

| **Mental health care arrangements for this patient** | |
|---|---|
| **1.** All mental health contacts for this patient have taken place within a primary care setting with no evidence of any contact with anyone outside of the immediate primary care team. | ☐ |
| **2.** In addition to primary care contacts this patient has received the majority of mental health care with a mental health specialist in a non-primary care setting (e.g. psychiatry out patients, CMHT). | ☐ |
| **3.** This patient has received a multi-professional approach to patient care: | |
| **a.** Where a general practitioner (GP) and at least one other health professional (e.g. CPN, psychologist, psychiatrist) has been involved in their care. | ☐ |
| **b.** Which has included a named primary care mental health worker sometimes known as graduate mental health worker. | ☐ |
| **c.** Which includes a secondary care worker (e.g. CPN) attached to your practice. | ☐ |
| **d.** Which includes a case manager* | ☐ |
| **4.** This patient has received scheduled patient follow-ups. This maybe one or more scheduled telephone or face to face follow-up appointments to provide specific interventions, facilitate treatment adherence, or monitor symptoms or adverse effects. | ☐ |
| **5.** Both the primary and secondary health care professionals caring for this patient have put in place specific mechanisms to improve inter-professional communication. These include team meetings, case conferences, individual consultation/supervision, shared medical records, and patient-specific written or verbal feedback between care-givers. | ☐ |

*A case manager provides proactive, coordination of care to people who have a complex mix of health and social care needs with the aim of preventing unnecessary admissions to hospital and providing care in the person's home or community setting and relieving carers of having to coordinate services and navigate a range of health and social care systems.

**44.** Are there any other issues related to this patient's health care that you think are relevant which we have not been able to capture within this questionnaire?

**45.** If there were questions / sections you found difficult to complete for this patient, please tell us which question / section and why?
