# Supplementary material for: Status of primary and secondary mental healthcare of people with severe mental illness: an epidemiological study from the UK PARTNERS2 programme
Source: BJPsych Open. 2021 Feb 15;7(2):e53. doi: 10.1192/bjo.2021.10 (PMC8058911; doi:10.1192/bjo.2021.10)
Supplement: Supplementary file 1 [file S2056472421000107sup001.zip › ONLINE_DETSecondaryCare.pdf]

PARTNERS2: ASSESSMENT OF LOCAL HEALTH CARE PATHWAYS AND CURRENT SERVICES FOR PEOPLE WITH SEVERE MENTAL ILLNESS (Secondary Care)

PATIENT DATA EXTRACTION FORM TO BE COMPLETED USING THE STUDY MANUAL

A: Your details

1. Name of data extractor:...

B: Patient ID and date of data extraction

2. Unique patient study No.: ☐☐☐ ☐

3. Date of data extraction ☐☐ / ☐☐ / ☐☐ ☐☐ **4**. Site No: ☐

Site number:
1 = Birmingham
2 = Devon
3 = Lancashire

5. Data extraction period:

From ☐☐ / ☐☐ / ☐☐ ☐☐ to ☐☐ / ☐☐ / ☐☐ ☐☐

6. GP practice code ☐ See manual for codes **7.** GP practice postcode: ☐☐☐☐ ☐☐☐

C: Patient demographic details

8.. Patient age (in years) at August 2014 ☐☐☐

9. Patient gender: ☐ 1 Male ☐ 2 Female ☐ 3 Transgender

10 Is the patient British? ☐ 1 Yes ☐ 2 No ☐ 99 Not recorded

11.. Patient ethnicity ☐

White
1 = White British
2 = White Irish
3 = Gypsy or Irish traveler
4 = White Other

Mixed/multiple ethnic groups
5 = White and black Caribbean
6 = White and black African
7 =White and black Asian
8 = Any other mixed/multiple Ethnicity background

Asian/Asian British
9 = Indian
10 = Pakistani
11 = Bangladeshi
12 = Chinese
13 = Any other Asian back ground

Black/African/Caribbean/Black British
14 = African
15 = Caribbean
16 = Any other black/African/Caribbean background

Other Ethnic Groups
17 = Arab
18 = Any other ethnic group – please specify:

…………………………………………………………………

99 = Not recorded

12. Usual Living situation: ☐

1 = Alone
2 = With parents
3 = With spouse/partner
4 = With spouse/partner & children
5 = With children – lone parent
6 = With other family
7 = with non-family
8 = Group home
9 = Other (Please specify)

…………………………………………….

99 = Not recorded

13. Does the patient have any children? If yes, please specify the number of children within each age range.

☐ 1 Yes ☐ 2 No ☐ 99 Not recorded

☐ 1 Under 18 ☐ 2 Over 18 ☐ 99 Not recorded

14. Has this patient been homeless in the last 5 years?

☐ 1 Yes ☐ 2 No ☐ 99 Not recorded

15. Current employment: ☐

1 = In full-time employment
2 = In part-time employment
3 = Self employed
4 = Not working (housewife/househusband)
5 = Not working - retired
6 = Not working – unemployed
7 = Not working – other (please specify) …………………..
8 = Voluntary work
9 = Full time carer
10 = Other (please specify) ………………………………………….
99 = Not recorded

16. Religion: ☐

1 = No religion
2 = Christian (E.g. Church of England, Catholic, Protestant and all other Christian denominations)
3 = Buddhist
4 = Hindu
5 = Jewish
6 = Muslim
7 = Sikh
8 = Any other religion please specify………………………………
99 = Not recorded

17. Does this patient **currently** receive any benefits (including permitted work, personal independence payments, employment support allowance, or disability allowance)?

☐ 1 Yes ☐ 2 No ☐ 99 Not recorded

18. Refugee status

☐ 1 Yes ☐ 2 No ☐ 99 Not recorded

19. Asylum seeker status

☐ 1 Yes ☐ 2 No ☐ 99 Not recorded

D : Patient health and medication details

20. What is this patient's current (most recent) serious mental illness diagnosis: ☐

0 = Not recorded
1 = Schizophrenia
2 = Schizotypal personality
3 = Persistent delusional Disorder
4 = Acute/Transient psychotic disorders
5 = Induced delusional disorder
6 = Schizoaffective disorders
7 = Manic Episodes
8 = Bipolar Disorder
9 = Severe Depression with Psychosis
10 = Schizotypal Personality
11 = Both schizophrenia and bipolar disorder
12 = Other (Please specify):
…………………………………………………
99 = Not recorded

21. Please provide as much additional details on diagnoses as reasonably possible (e.g. exact diagnosis, previous diagnoses, year of first serious mental illness diagnosis)

…………………………………………………………......

…………………………………………………………...…

…………………………………………………………

22. What date was this diagnosis (Q20) first made? ☐☐ / ☐☐ / ☐☐☐☐ or tick ☐ for not recorded

23. Is this date exact or an estimate? 1 Exact ☐ 2 Estimate ☐

24. Is there evidence of a dual diagnosis (i.e. a substance misuse or alcohol problem) for this patient?

☐ 1 Yes ☐ 2 No ☐ 99 Not recorded

☐ 1 Current ☐ 2 Past ☐ 99 Not recorded

25. Have any cluster categories been recorded within the data extraction period?

☐ 1 Yes ☐ 2 No ☐ 99 Not recorded

If yes, please complete the table below. If no, go to question 26.

Box 1

0 None recorded/not been assigned a cluster

1 Common mental health problems (low severity)

2 Common mental health problems (low severity with greater need)

3 Non-psychotic (moderate severity)

4 Non-psychotic (severe)

5 Non-psychotic (very severe)

6 Non-psychotic disorders of overvalued ideas

7 Enduring non-psychotic disorders (high disability)

8 Non psychotic chaotic and challenging disorders

9 Blank cluster

10 First episode in psychosis

11 Ongoing recurrent psychosis (low symptoms)

12 Ongoing or recurrent psychosis (high disability)

13 Ongoing or recurrent psychosis (high symptom and disability)

14 Psychotic crisis

15 Severe psychotic depression

16 Dual diagnosis – substance misuse and mental illness

17 Psychosis and affective disorder – difficult to engage

18 Cognitive impairment (low need)

19 Cognitive impairment or dementia (moderate need)

20 Cognitive impairment or dementia (high need)

21 Cognitive impairment (high physical need or engagement)

Please refer to the manual for guidance on the required timings for each of the clusters

Box 2 - Within required timing for reassessment (see manual)
1 = Yes
2 = No

Cluster category (Box 1)	Date recorded (dd/mm/yyyy)	Within required timing (Box 2)
	_ _/_ _/_ _ _ _	
	_ _/_ _/_ _ _ _	
	_ _/_ _/_ _ _ _	
	_ _/_ _/_ _ _ _	
	_ _/_ _/_ _ _ _	
	_ _/_ _/_ _ _ _	
	_ _/_ _/_ _ _ _	
	_ _/_ _/_ _ _ _	
	_ _/_ _/_ _ _ _	
	_ _/_ _/_ _ _ _	
	_ _/_ _/_ _ _ _	
	_ _/_ _/_ _ _ _	

	_ _/_ _/_ _ _ _	
	_ _/_ _/_ _ _ _	
	_ _/_ _/_ _ _ _	
	_ _/_ _/_ _ _ _	
	_ _/_ _/_ _ _ _	
	_ _/_ _/_ _ _ _	
	_ _/_ _/_ _ _ _	
	_ _/_ _/_ _ _ _	
	_ _/_ _/_ _ _ _	
	_ _/_ _/_ _ _ _	
	_ _/_ _/_ _ _ _	
	_ _/_ _/_ _ _ _	
	_ _/_ _/_ _ _ _	
	_ _/_ _/_ _ _ _	
	_ _/_ _/_ _ _ _	

Please continue on additional sheets if necessary

26. Please tick any major health morbidities recorded in the patient case notes:

☐ 1 Diabetes	☐ 8 Ischaemic heart disease	☐ 15 Cancer
☐ 2 Asthma	☐ 9 Heart failure	☐ 16 Osteoarthritis
☐ 3 COPD	☐ 10 Chronic kidney disease	☐ 17 Obesity
☐ 4 Epilepsy	☐ 11 Depression	☐ 18 Visual problems
☐ 5 Hypertension	☐ 12 Learning disability	☐ 19 Anxiety
☐ 6 Stroke	☐ 13 Hearing problems	☐ 20 None
☐ 7 Thyroid disorder	☐ 14 Rheumatoid arthritis	☐ 21 Other (please specify)

....
....
....
....
....
....
....
....
....
....

27. Please record all current mental health medications including all oral and depot medications using the codes in box 1.
Please see Appendix 1 of the instruction manual for a list of medications by generic name.

Box 1: Medication type

1 = Antipsychotic (conventional)
2 = Antipsychotic (atypical)
3 = Bipolar disorder medications
4 = Antidepressant medications
5 = Antianxiety medications
6 = Other mental health meds
7 = Any other medication
99 – Data not recorded/not known/missing
0 = No prescription

Medication type (Box 1)	Name of medication	Date of first documented prescription for this drug dd/mm/yyyy<br><br>Or please provide a rough estimate if available – please note if this is an estimate	Ongoing?<br>1 = Yes<br>2 = No<br>99 = Data not recorded/not known/missing

Please continue on additional sheets if necessary

28. Has the patient had a blood pressure, bloods, BMI, cholesterol or blood glucose levels checked and recorded within the data extraction period?

☐ 1 Yes ☐ 2 No ☐ 99 Not recorded

If yes, please record in the table below.

Box 1
By recommended action we mean the action that is recommended in the UK Lester adaptation - Positive Cardiometabolic Health Resource (Appendix 2)

1 = Yes – recommended action taken as a result of the abnormal reading
2 = No action taken
3 = Not applicable as results were in range
99 = Not recorded/unclear/data missing
4 = Recommended action taken as part of general physical health check

**Date recorded Dd/mm/yyyy**	Please document BP (systolic/ diastolic) or record 99 if not taken	If BP was abnormal (see appendix) was the recommended action taken and documented in the notes (Box1)		**Date recorded Dd/mm/yyyy**	Please document BMI/waist circumference or record 99 if not taken	If BMI was abnormal (see appendix) was the recommended action taken and documented in the notes (Box 1)		**Date recorded Dd/mm/yyyy**	Bloods taken 1 = yes 2 = no 99 =not recorded		**Date recorded Dd/mm/yyyy**	Please document cholesterol or record 99 if not taken	If cholesterol was abnormal (see appendix) was the recommended action taken and documented in the notes (Box 1)		**Date recorded Dd/mm/yyyy**	Please document HbA1c levels or record 99 if not taken (Box 1)	If HbA1c was abnormal (see appendix) was the recommended action taken and documented in the notes

Please continue on additional sheets if necessary

29. Please fill in the following table for the most recent date that the health checks were performed, or record 99 in date column if not recorded.

00 = Never
99 = Not recorded

Please record the most recent date that the following health checks were recorded	Date (dd/mm/yyyy)
LUNSERS/Other side effects rating scale	
Eyes tested	
ECG	
Visited the dentist	
Cervical smear	
Mammogram (females over 50 only)	

E: Lifestyle

30. Is this patient a smoker?

☐ 1 Yes ☐ 2 No ☐ 3 Ex- smoker ☐ 99 Not recorded

☐ 1 1-10 ☐ 2 11-20 ☐ 3 21-30 ☐ 4 30+ per day ☐ 99 Not recorded

If yes, please complete question 31. If no, please skip to question 32.

31. Has the patient had any help to stop smoking between the data extraction dates? e.g. referred to smoking cessation or prescribed nicotine replacement therapy?

☐ 1 Yes ☐ 2 No ☐ 3 Patient declined ☐ 99 Not recorded

32. Is there any evidence that this patient drank alcohol between the data extraction dates?

☐ 1 Yes ☐ 2 No ☐ 99 Not recorded

If yes, please document the most recent level of alcohol consumption **per week** documented in the notes.

☐ 1 1-5 units ☐ 2 6-10 units ☐ 3 11-15 units

☐ 4 16-20 units ☐ 5 21-25 units ☐ 6 26-30 units

☐ 7 31-35units ☐ 8 >35 units ☐ 99 Not recorded

☐ 9 Other amount/other frequency (please specify)………………………………….……..……………

33. Is there any evidence that this patient was taking/has taken recreational/non-prescription drugs between the data extraction dates?

☐ 1 Yes ☐ 2 No ☐ 99 Not recorded

- ☐ 1 Ecstasy
- ☐ 2 LSD
- ☐ 3 Heroin
- ☐ 4 Cocaine
- ☐ 5 Crack Cocaine
- ☐ 6 Magic Mushrooms
- ☐ 7 Methadone
- ☐ 8 Meth Amphetamine (Crystal Meth)
- ☐ 9 Amphetamines
- ☐ 10 Cannabis
- ☐ 11 Codeine
- ☐ 12 Methylphenidate
- ☐ 13 Pholcodine
- ☐ 14 Other – Please specify:
- ☐ 99 Not recorded

……………………………………………

……………………………………………

……………………………………………

……………………………………………

F: Service use

34. Have there been any hospital admissions between the data extraction dates? Please include respite care here. Please also look at the free text in the patient's records.

☐ $_1$Yes ☐ $_2$No ☐ $_{99}$Not recorded

If yes, please complete the table below.

Box 1: Route of admission

1 = A&E
2 = GP
3 = Crisis team
4 = Other (please specify)
99 = Not recorded/not known

Box 2: Specialty

1 = Mental health (voluntary)
2 = Mental health (Compulsory)
3 = Cardiology
4 = Respiratory
5 = Ophthalmology
6 = ENT
7 = Gastroenterology
8 = Neurology
9 = Orthopedics
10 = Physiotherapy
11 = Cancer care
12 = Palliative care
13 = Other surgery
14 = Dermatology
15 = Stroke
16 = Gynecology
17 = Chiropody
18 = Private hospital
19 = Respite
20 = Other (please specify)
99 = Not recorded/not known

Box 3 Reason for admission

1 = Suicide attempt / overdose
2 = Self harm
3 = Alcohol / substance abuse
4 = Accidental injury
5 = psychiatric help
6 = Physical health problem
7 = Prescription run out
8 = Diagnosis
9 = Specialist investigation
10 = Physiotherapy
11 = Antenatal care
12= Other/unsure which category (please specify)
99 = Not recorded/not known

Date admitted (dd/mm/yyyy)	Date discharged (dd/mm/yyyy)<br><br>If patient is still in hospital indicate by entering 88/88/88	Route of admission (Box 1)	Specialty (Box 2)	Reason for admission (Box 3)

Please continue on additional sheets if necessary

35. Excluding inpatient admissions and mental health services, has there been any referrals or visits to hospital, A&E visits, walk in Centre visits or out of hours primary care contacts within the data extraction period?

Yes ☐ No ☐ Not Recorded ☐

If yes, please complete the table below.

**Type of contact (Box 1)**
1 = Referrals to hospital (not admission)
2 = A&E visits
3 = Walk-in-Centre visits
4 = Out of hours primary care contact
5 = Pharmacy
6 = Called Ambulance
7 = Other (please specify in table)
99 = Not recorded/Not known

**Box 2: Specialty**	
1 = Cardiology	10 = Palliative care
2 = Respiratory	11 = Other surgery
3 = Ophthalmology	12 = Dermatology
4 = ENT	13 = Stroke
5 = Gastroenterology	14 = Gynecology
6 = Neurology	15 = Chiropody
7 = Orthopedics	16 = Other (please specify in table)
8 = Physiotherapy	99 = Not recorded/not known
9 = Cancer care	

**Box 3 Reason for contact**
1 = Suicide attempt / overdose
2 = Self harm
3 = Alcohol / substance abuse
4 = Accidental injury
5 = psychiatric help
6 = Physical health problem
7 = Prescription run out
8 = Diagnosis
9 = Specialist investigation
10 = Physiotherapy
11 = Antenatal care
12= Other/unsure which category (please specify)
99 = Not recorded

**Type of contact (Box 1)**	**Specialty**	**Date of contact (dd/mm/yyyy)**	**Reason for contact (Box 3)**	**Single visit (Yes/no)**	**If more than 1 visit per referral please give total number of visits**

Please continue on additional sheets if necessary.

36. Mental health service use within the data extraction period.

Please complete the following table regarding **all contacts** the patient's contact with mental health services with the data extraction period (please see the manual for further detail).

Or if non recorded please tick here ☐

Mental health service referred to/seen by (Box 1)
1= CMHT (or equivalent) – general
2= CMHT (or equivalent) – depot injection
3 = CMHT (or equivalent) – home depot
4= CMHT (or equivalent) – medication clinic
5 = Outpatient psychiatry
6 = Home treatment teams/crisis resolution
7 = Day care
8 = Rehabilitation/Recovery
9 = Assertive community treatment
10 = Social Services (please specify type)
11 = Forensic
12 = Back to primary care
13 = Early interventions services
14 = CBT
15 = Family Therapy
16 = Other types of psychotherapy
17 = Crisis housing
18 = Voluntary organisation support service
19 = Psychologist
20 = Drugs and alcohol services
21 = Homelessness services
22 = IAPT
23 = Counselling
24 = Chaplaincy
25 = Art therapy
26 = Support groups
27 = Out of hours crisis/support line
28 = Other/unsure which category (please specify)
99 = Not recorded

Which professional has the patient seen?
1 = Social worker
2 = Social work assistant
3 = Nurse
4 = Occupational therapist
5 = Occupational therapy assistant
6 = Clinical psychologist
7 = Assistant psychologist
8 = Consultant psychiatrist
9 = Trust/staff grade psychiatrist
10 = Support worker/STR worker/HCA
11 = Peer worker
12 = Voluntary sector worker
13 = Student
14 = Administrator 15 = police doctor
16= Other/Unsure which category (please specify)
17 = Mental health worker in secondary care
99 = Not recorded

Reason for discharge
1 = Did not attend
2 = Lost to follow up
3 = Patient request
4 = Team felt not needed
5 = Other (please specify)
99 = Not recorded

Method of contact
1 = Telephone
2 = Letter
3 = Face to face
4 = Other (please specify)
99 = Not recoded

Self referral
1 = Yes
2 = No
99 = Not recorded

Place of contact
1 = Home visit
2 = Clinic outpatients
3 = Community venue
4 = Voluntary organisation site
5 = Contact call to service user (outgoing)
6 = Contact call from service user (Incoming)
7 = Third party contact about the service user (incoming)
8 = Third party contact about the service user (outgoing)
9 = No access visit/Did not attend/failed contact
10 = Other (please specify)
11 = Police custody
99 = Not recorded

Care coordinator?
1 = Yes 99 = Not recorded
2 = No

Date of referral NB this may be before the data extraction period starts (dd/mm/yyyy)	Date seen (dd/mm/yyyy)	Service	If box 1 = 10 please type of social service<br><br>If box 1 = 28, please specify	Self referral?	Method of contact if recorded?	Which professional?	Is this professional the care-coordinator/ case manager?	Place of contact?	Date of discharge (dd/mm/yyyy)	Reason for discharge

Date of referral NB this may be before the data extraction period starts (dd/mm/yyyy)	Date seen (dd/mm/yyyy)	Service	If box 1 = 10 please type of social service If box 1 = 28 please specify	Self referral	Method of contact	Which professional?	Is this professional the care coordinator/ case manager?	Place of contact?	Date of discharge (dd/mm/yyyy)	Reason for discharge

Date of referral NB this may be before the data extraction period starts (dd/mm/yyyy)	Date seen (dd/mm/yyyy)	Service	If box 1 = 10 please type of social service<br><br>If box 1 = 28, please specify	Self referral	Method of contact	Which professional?	Is this professional the care coordinator/ case manager?	Place of contact?	Date of discharge (dd/mm/yyyy)	Reason for discharge

Please continue on additional sheets if necessary.

Q36b. Within the CMHT (or equivalent) has there been any transfer between the 'sub-teams':

Codes:
1 = West Lancashire (when merged as a CMHT)
2 = West Lancashire Recovery Team
3 = West Lancashire CCTT
4 = A Devon Community MH Recovery Team
5= A Devon Community Psychosis Recovery Team
6 = A Devon Recovery and Independent Living Team
7. Psychosis and Recovery Team (South and West Devon)
8. Mental Health and Recovery (South and West Devon)
9 = Birmingham – name of team
10 = Birmingham -name of team
11 = Other – please specify in table
99 = Not recorded/unsure which team

Ref date	Team	Discharge date

37. Has this person had a named care co-coordinator/case manager for any period during **the data extraction periods**?

- ☐ 1 Yes → Please complete question 38
- ☐ 2 No → Go to question 40
- ☐ 99 Not recorded → Go to question 40

**Box 1 Care coordinators/case managers professional group**
1 = Social worker
2 = CPN
3 = Occupational therapist
4 = Clinical psychologist
5 = Consultant psychiatrist
6 = Mental health worker in secondary care
7 = Junior doctor
8 = Staff grade psychiatrist
9 = Mental Health Practitioner
10 = Recovery Practitioner
11 = Other or unsure which category (please specify in table)
12 = Period with no care coordinator/case manager
99= Not recorded

38. Please specify the professional groups and number of care coordinators/case managers for each year using the codes above

Year 1		Year 2	
Professional Group	Total number from this professional group	Professional Group	Total number from this professional group
Total for data extraction period = ☐			

39. If there has been a single named care coordinator/case manager for both time periods, was the care coordinator the same person for each time period?

☐ 1 Yes ☐ 2 No ☐ 99 Not recorded

40. Has this patient had a named support worker/STR worker for any period during the data extraction dates?

- ☐ 1 Yes → Go to question 41
- ☐ 2 No → Go to question 44
- ☐ 99 Not recorded → Go to question 44

41. How many different named support workers/STR workers has the patient been allocated between the data extraction dates? ☐☐

42. How many different named support workers/STR workers has the patient been allocated in the first year of the data extraction period? ☐☐

43. How many different named support workers/STR workers has the patient been allocated in the second year of data collection? ☐☐

44. How many different psychiatrists has this patient seen within the data extraction dates**?** ☐☐

45. How many different psychiatrists has the service user seen in the first year of the data extraction period**?** ☐☐

46. How many different psychiatrists has the service user seen within the second year of data extraction? ☐☐

47. Has this patient been subject to a community treatment order within the data extraction period?

☐ 1 Yes ☐ 2 No ☐ 99 Not recorded

48. Has the patient been under section 117 within the data extraction period?

☐ 1 Yes ☐ 2 No ☐ 99 Not recorded

49. Have there been any CPA/review meetings held between the data extraction dates?

☐ 1Yes ☐ 2No ☐ 99Not Recorded

If yes please complete the table below for all CPA or review meetings held between **the data extraction dates**:

1 = Yes
2 = No
99 = Not recorded

Date (dd/mm/yyyy)	Evidence of CPA documents given to			Attended CPA/review				There is evidence the patient has been involved in their care plan development
	Patient?	Sent to GP?	Communicated to care giver?	Patient	GP	Caregiver (Next of kin/carer/key worker from voluntary sector)	Psychiatrist	

Please continue on additional sheets if necessary

50. Are there any other issues related to this patient's health care that you think are relevant which we have not been able to capture within this questionnaire? Please provide a short overview about the patient here – see manual for further details

51. If there were questions/sections you found difficult to complete for this patient, please tell us which question/section and why?

Additional sheets used: ☐ (Please attach)
