# Supplementary material for: Status of primary and secondary mental healthcare of people with severe mental illness: an epidemiological study from the UK PARTNERS2 programme
Source: BJPsych Open. 2021 Feb 15;7(2):e53. doi: 10.1192/bjo.2021.10 (PMC8058911; doi:10.1192/bjo.2021.10)
Supplement: Supplementary file 1 [file S2056472421000107sup001.zip › PARTNERS2_WS1_ONLINE_tables_and_figure_130920.docx]

# ONLINE FIGURE & TABLES

**Figure A: Data from Partners2 Workstream 1 study presented in this paper †**

**
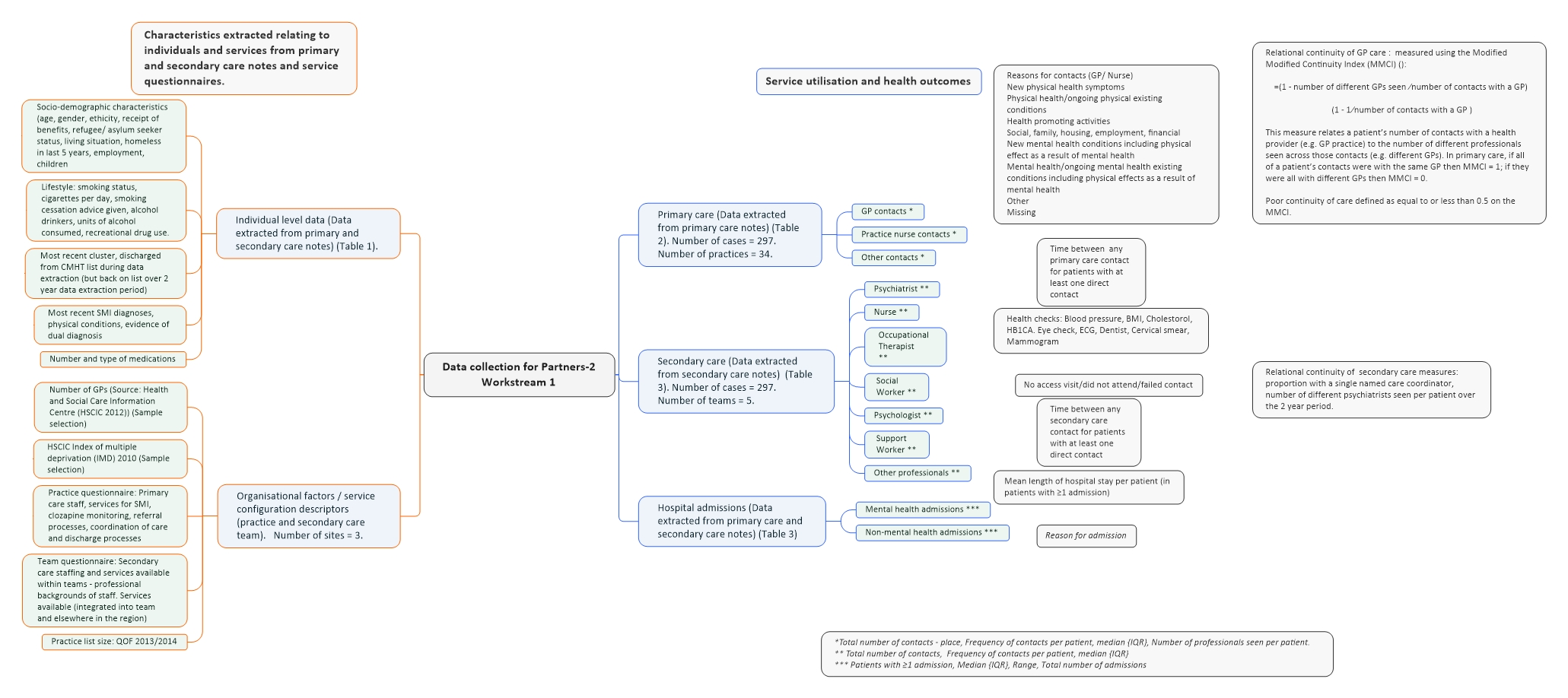
**

**†**In order to review the case notes for the same person from both secondary mental health care and primary care medical records and for the same data extraction period, the secondary care data collector sent the minimum essential information to the primary care data collector via secure email (NHS number, GP practice and unique identifiable number). This information was sent via a secure and approved email service connection using NHSmail

#

**Table A: Practice list size, number of GPs and index of multiple deprivation in participating practices compared with practices not included**

|  | **Birmingham** | | **Devon** | | **Lancashire ^1^** | | **All participating**  **practices^2^** | **Other Practices in England** |
| --- | --- | --- | --- | --- | --- | --- | --- | --- |
|  | Participating^2^ | Declined | Participating^2^ | Declined | Participating^2^ | Declined |  |  |
|  | N=4 | N=11 | N=18 | N=13 | N=12 | N=11 | N=34 | N=8366 |
| **List size^$^**  Mean [SD]  95% CI | 5309 [4599]  (0 to 12626) | 5528 [3111]  (3438 to 7618) | 8617 [4154]  (6552 to 10683) | 6822 [2328]  (5415 to 8229) | 5985 [2911]  (4029 to 7940) | 4192 [2275]  (2663 to 5720) | 7339 [3982] | 7086 [4366] |
| Median  {IQR} | 3534  {2275 to 8342} | 4822  {3453 to 7114} | 9404  {4966 to 11571} | 7120  {4420 to 8144} | 5713  {2777 to 7809} | 2842  {2337 to 6328} | 6982  {3763 to 10468} | 6247  {3727 to 9592} |
| **Number of GPS ^*^**  Mean [SD]  95% CI | 3.3 [2.2]  (0 to 6.8) | 2 [1.4]  (1.1 to 3.0) | 5.1 [2.3]  (3.9 to 6.2) | 4.9 [2.4]  (3.5 to 6.4) | 2.9 [1.6]  (1.8 to 4.0) | 2.1 [1.2]  (1.3 to 2.9) | 4.1 [2.3]  (3.3 to 4.9) | 3.4 [2.4]  (3.3 to 3.4) |
| **IMD 2010^**^**  1  (least deprived) | 0 (-)  (0% to 60%) | 0 (-)  (0% to 28%) | 4 (22%)  (6% to 48%) | 3 (23%)  (5% to 54%) | 3 (27%)  (6% to 61%) | 4 (36%)  (11% to 69%) | 7 (21%) | 2053 (25%) |
| 2 | 0 (-)  (0% to 60%) | 0 (-)  (0% to 28%) | 13 (72%)  (47% to 90%) | 8 (62%)  (32% to 86%) | 5 (45%)  (17% to 77%) | 2 (18%)  (2% to 52%) | 18 (55%) | 2037 (25%) |
| 3 | 0 (-)  (0% to 60%) | 2 (18%)  (2% to 52%) | 1 (6%)  (0.1% to 27%) | 1 (8%)  (2% to 36%) | 1 (9%)  (0.2% to 42%) | 4 (36%)  (11% to 69%) | 2 (6%) | 2062 (25%) |
| 4 (most deprived) | 4 (100%)  (40% to 100%) | 9 (82%)  (48% to 98%) | 0 (-)  (0% to 19%) | 1 (8%)  (2% to 36%) | 2 (18%)  (2% to 52%) | 1 (9%)  (0.2% to 42%) | 6 (18%) | 2045 (25%) |

^1^Notes were extracted from two teams operating from the same base in Lancashire. At the time of data collection these were a complex community treatment team (CCTT) and a recovery team. Teams have since merged into a CMHT.

^2^After a defined cut- off date practices were defined as participating if they consented or ‘did not respond’.

$ Source: QOF 2013/2014

* Source: HSCIC 2012

**Source: HSCIC IMD 2010: index of multiple deprivation 2010. Quartiles based on General Practice average weighted scores.

**Table B: Reasons for primary care consultations**

| ***Reason for contact (all primary care professionals)*** | *n_1_ (%); n_2_ (%)* |
| --- | --- |
| Mental health/ongoing mental health existing conditions including physical effects as a result of mental health | 1457 (34); 204 (69) |
| Physical health/ongoing physical existing conditions | 1406 (33); 207 (70) |
| New physical health symptoms | 712 (17); 177 (60) |
|  |  |
| Health promoting activities | 372 (9); 148 (50) |
| Social, family, housing, employment, financial | 43 (1); 24 (8) |
| New mental health conditions including physical effect as a result of mental health | 25 (1); 19 (6) |
|  |  |
| Other | 262 (6); 68 (23) |
| Missing | 10 (<1); 8 (3) |

*n1 = number of contacts
n2 = number of patients with ≥1 contact*
